# Hemodynamic effects of lung recruitment maneuvers in acute respiratory distress syndrome

**DOI:** 10.1186/s12890-017-0369-7

**Published:** 2017-02-08

**Authors:** Anup Das, Mainul Haque, Marc Chikhani, Oana Cole, Wenfei Wang, Jonathan G. Hardman, Declan G. Bates

**Affiliations:** 1 School of Engineering, University of Warwick, Nottingham, CV4 7AL UK; 2 Division of Clinical Neuroscience, School of Medicine, University of Nottingham, Nottingham, NG7 2UH UK; 3 Nottingham University Hospitals NHS Trust, Nottingham, NG7 2UH UK

**Keywords:** Acute respiratory distress syndrome, Recruitment maneuvers, Positive end expiratory pressure, Cardiac output, Computational modelling, Oxygen delivery, Carbon dioxide clearance, Strain, Mechanical ventilation

## Abstract

**Background:**

Clinical trials have, so far, failed to establish clear beneficial outcomes of recruitment maneuvers (RMs) on patient mortality in acute respiratory distress syndrome (ARDS), and the effects of RMs on the cardiovascular system remain poorly understood.

**Methods:**

A computational model with highly integrated pulmonary and cardiovascular systems was configured to replicate static and dynamic cardio-pulmonary data from clinical trials. Recruitment maneuvers (RMs) were executed in 23 individual *in-silico* patients with varying levels of ARDS severity and initial cardiac output. Multiple clinical variables were recorded and analyzed, including arterial oxygenation, cardiac output, peripheral oxygen delivery and alveolar strain.

**Results:**

The maximal recruitment strategy (MRS) maneuver, which implements gradual increments of positive end expiratory pressure (PEEP) followed by PEEP titration, produced improvements in PF ratio, carbon dioxide elimination and dynamic strain in all 23 *in-silico* patients considered. Reduced cardiac output in the moderate and mild *in silico* ARDS patients produced significant drops in oxygen delivery during the RM (average decrease of 423 ml min^−1^ and 526 ml min^−1^, respectively). In the *in-silico* patients with severe ARDS, however, significantly improved gas-exchange led to an average increase of 89 ml min^−1^ in oxygen delivery during the RM, despite a simultaneous fall in cardiac output of more than 3 l min^−1^ on average. Post RM increases in oxygen delivery were observed only for the *in silico* patients with severe ARDS. In patients with high baseline cardiac outputs (>6.5 l min^−1^), oxygen delivery never fell below 700 ml min^−1^.

**Conclusions:**

Our results support the hypothesis that patients with severe ARDS and significant numbers of alveolar units available for recruitment may benefit more from RMs. Our results also indicate that a higher than normal initial cardiac output may provide protection against the potentially negative effects of high intrathoracic pressures associated with RMs on cardiac function. Results from *in silico* patients with mild or moderate ARDS suggest that the detrimental effects of RMs on cardiac output can potentially outweigh the positive effects of alveolar recruitment on oxygenation, resulting in overall reductions in tissue oxygen delivery.

**Electronic supplementary material:**

The online version of this article (doi:10.1186/s12890-017-0369-7) contains supplementary material, which is available to authorized users.

## Background

Recruitment maneuvers (RMs) are used as a strategy to improve oxygenation and reduce the risk of atelectrauma in ARDS patients by re-opening and stabilising collapsed lung regions [1]. Several RMs have so far been proposed, including sustained inflations with continuous positive airway pressure of 35–50 cm H_2_0 for 20–40 s [2], incremental peak inspiratory pressures [3], lower tidal volumes (with sighs), intermittent sighs [4], stepwise increments in positive end-expiratory pressure (PEEP) [5], and slow increases of inspiratory pressure to 40 cm H_2_O [6]. Despite numerous studies, there is still little conclusive evidence that RMs improve overall outcomes (including mortality) in critically ill patients [4, 7, 8]. The consensus is that RMs should be considered on an individual basis, but the optimal pressure, duration and frequency of RMs remain to be determined, and few guidelines are available to enable effective patient stratification.

Increased intrathoracic pressures (P_IT_) produced by RMs significantly affect left ventricular (LV) preload, right ventricular (RV) afterload and biventricular compliance [9]. Right ventricular preload is also affected by the impairment of the right atrium and by increased resistance to systemic venous return. Increase in P_IT_ reduces the pressure gradient between the systemic venous pressure and the RV diastolic pressure, reducing venous return, decreasing RV filling and consequently decreasing stroke volume (SV) and decreasing inflow to the left ventricle [10]. This passive relationship between RV and LV is compounded by the direct effects of raised P_IT_ [9] on the ventricular walls (splinting) as well as the potential for intraventricular septum shift (ventricular interdependence) [11]. The consequences of these complex relationships affecting RV/LV function and heart-lung interaction are difficult to quantify or investigate in the clinical environment. Reliably evaluating the relative effectiveness of different RMs in clinical studies is also extremely challenging, since it is difficult to isolate the effects of ventilatory strategies, and because different RMs cannot be applied to the same patient simultaneously.

In contrast, *in silico* models of individualised patient and disease pathology allow different RMs to be applied to the same patient with exactly the same baseline pathophysiology, in order to understand their mode of action and quantitatively compare their effectiveness in different scenarios. Previous computational modelling studies have shown the potential of this approach to add significantly to our understanding of cardiopulmonary pathophysiology [12, 13] and the mechanisms associated with alveolar recruitment [13–16].

## Methods

### Computational model

**Fig. 1:**
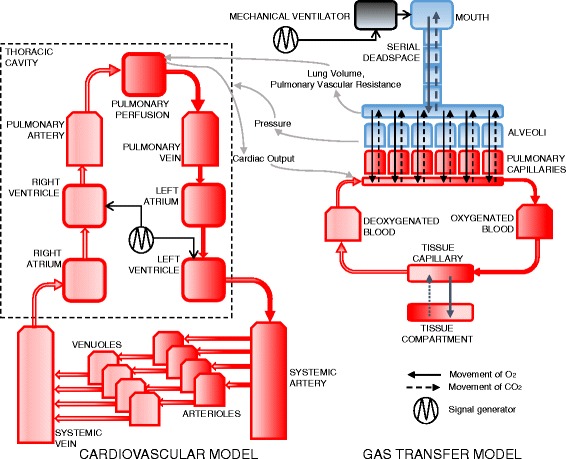
Architecture of the integrated cardiopulmonary model

Our study employs a highly integrated computer simulation model of the pulmonary and cardiovascular systems that has recently been developed by our group [17–19]. The model architecture and its main components are depicted in Fig. 1. The pulmonary model includes 100 independently configured alveolar compartments, multi-compartmental gas-exchange, viscoelastic compliance behaviour, interdependent blood-gas solubilities and haemoglobin behaviour and heterogeneous distributions of pulmonary ventilation and perfusion. The ability of this model to accurately represent multiple aspects of pulmonary pathophysiology have been validated in a number of previous studies [17, 20–22]. This model was integrated with a dynamic, contractile cardiovascular model with 19-compartments, pulsatile blood flow and ventilation-affected, trans-alveolar blood-flow. The cardiac section of the model consists of two contractile ventricles, with atria modelled as non-contractile, low-resistance, high-compliance compartments.

Cardiopulmonary interactions are modelled in a number of ways. Ventricular contractility is modelled as a truncated sine-wave that varies ventricular elastance over time [23]. Intrapulmonary pressure is transmitted variably across ventricular walls (depending on ventricular stiffness) such that lung inflation “splints” the ventricles; transmitting intrathoracic pressure to the intraventricular and intravascular spaces. Trans-alveolar blood flow is governed by pulmonary artery pressure, and by independent trans-alveolar vascular resistance; this resistance is affected dynamically in each alveolar compartment by alveolar volume (causing longitudinal stretch) and pressure (causing axial compression).

The mathematical principles and equations underpinning the model are explained in detail in the Additional file 1.

### Measurements

To observe the hemodynamic effects of interest, the following values were recorded: cardiac output (CO), right ventricle end diastolic volume (RVEDV), right ventricle end systolic volume (RVESV), mean arterial pressure (MAP), and mean pulmonary artery pressure (MPAP). Other parameters recorded from the model included: arterial oxygen tension (PaO_2_), arterial carbon dioxide tension (PaCO_2_), arterial pH (pH_a_), arterial and mixed venous oxygen saturation (SaO_2_ and SvO_2_, respectively), static lung compliance (Cstat), plateau pressure (Pplat), volume of individual alveolar compartments at end of inspiration and end of expiration (V_alv_insp_ and V_alv_exp_, respectively), and pressure of individual compartments at end of inspiration and end of expiration (P_alv_insp_ and P_alv_exp_, respectively). Recruitment was calculated as the fraction of alveoli receiving non-zero ventilation. The strain on the lung is given as both dynamic and static [24]. The dynamic strain is calculated as ∆V/V_frc_, where V_frc_ is V_alv_exp_ at PEEP = 0 and ∆V = V_alv_insp_-V_alv_exp_. Static strain is calculated as V_alv_exp_/V_frc_. All parameters were recorded every 10 milliseconds and the plots have been generated with mean values taken over a duration of 1 s.

### Patients datasets

Two ARDS patient datasets were selected from the published literature, based on their inclusion of hemodynamic responses to changes in mechanical ventilation (see Table 1 (for first datas﻿et) and Table 2 (for the second dataset).

**Table 1:** Results of fitting the model to ARDS patient data of PaO_2_ and PaCO_2_

		Moderate ARDS, High CO [25]		Moderate ARDS, Normal CO [26]		Severe ARDS, High CO [27]	
Parameters obtained from data	CO (l min^−1^)	8		4.09		7.3	
	F_I_O_2_	0.5		0.45		1	
	Vt (ml kg^−1^)	12		10		10	
	PEEP (cm H_2_O)	0		0		0	
Parameters determined by optimization^a^	VR (b min^−1^)	12		10		10	
	Duty Cycle	0.33		0.35		0.46	
	RQ	0.9		0.9		0.7	
	VO_2_ (ml min^−1^)	307		303		306	
	Hb (g dl^−1^)	9.9		14.5		10.5	
		Data	Model	Data	Model	Data	Model
Results of fitting the model to the data	PaO_2_ (kpa)	10.6	11.2	10	10.8	6.6	7.5
	PaCO_2_ (kpa)	5	4.4	5.3	5.2	3.7	4.3
Other results	PvO_2_ (kpa)	NA	4.6	NA	4.4	NA	4.1
	Shunt Fraction (%)	NA	22	NA	16	NA	44

List of Abbreviations *CO* cardiac output, *FiO*_2_ fraction of O_2_ in inspired gas, *Vt* tidal volume, *VR* ventilator rate, *PEEP* positive end expiratory pressure, *IE* inspiratory to expiratory ratio, *RQ* respiratory quotient, *VO*_2_ oxygen consumption, *TOP* threshold opening pressure, *S* alveolar stiffness factor, *Pext* extrinsic pressure, *Hb* hemoglobin in blood, *PaO*_2_ arterial oxygen tension, *PaCO*_2_ arterial carbon dioxide tension, *PvO*_2_ mixed venous oxygen tension, shunt fraction

^a^Optimization methodology and parameter ranges given in Additional file 1

**Table 2:** Results of fitting the model to 20 ARDS patient data of PaO_2_, PaCO_2_ and Cstat at baseline

		All Patients		Severe ARDS		Moderate ARDS		Mild ARDS	
	*n*	20		11		6		3	
	Vt (ml kg^−1^)	6							
	VR (b min^−1^)	12							
	PEEP (cm H_2_O)	10							
	Ventilation mode	Volume controlled							
	F_I_O_2_	1							
	HR (bpm)	100							
		mean	sd	mean	sd	mean	sd	mean	sd
Parameters determined by optimization^a^	CI (l min^−1^ m^−2^)	5.3	0.5	5.0	0.4	5.6	0.5	6.0	0.1
	RQ	0.8	0.1	0.8	0.1	0.8	0.1	0.8	0.1
	VO_2_ (ml min^−1^)	304.4	6.3	305.8	3.8	303.8	8.0	300.3	10.6
	Duty Cycle	0.4	0.0	0.4	0.0	0.4	0.0	0.4	0.0
	Hb (g l^−1^)	110.5	39.5	92.5	32.6	115.8	33.9	165.7	13.5
Results of fitting the model to the data	PaO2 (mm Hg)	120.9	73.2	68.6	10.6	149.5	36.1	255.3	49.7
	Cstat (ml/cm H_2_O)	25.0	6.4	22.0	4.5	27.3	4.5	31.7	10.1
	PaCO2 (mmHg)	61.2	3.6	59.3	2.9	62.3	3.3	65.7	1.2
Other results	Shunt Fraction (%)	37.6	12.3	46.5	5.0	30.8	5.5	18.7	11.7
	Pplat (cm H_2_O)	27.4	4.1	29.4	4.1	25.3	2.1	24.0	4.4
	TOP (cm H_2_O)	21.6	2.4	22.3	2.8	21.0	2.0	20.0	0.0

List of Abbreviations *CI* cardiac index, *FiO*_2_ fraction of O_2_ in inspired gas, *Vt* tidal volume, *VR* ventilator rate, *PEEP* positive end expiratory pressure, *IE* inspiratory to expiratory ratio, *RQ* respiratory quotient, *VO*_2_ oxygen consumption, *TOP* threshold opening pressure, *S* alveolar stiffness factor, *Pext* extrinsic pressure, *Hb* hemoglobin in blood, *PaO*_2_ arterial oxygen tension, *Cstat* static compliance, *PaCO*_2_ arterial carbon dioxide tension, *PvO*_2_ mixed venous oxygen tension, *TOP* threshold opening pressures, *Pplat* plateau pressure

^a^Optimization methodology and parameter ranges given in Additional file 1

**Fig. 2:**
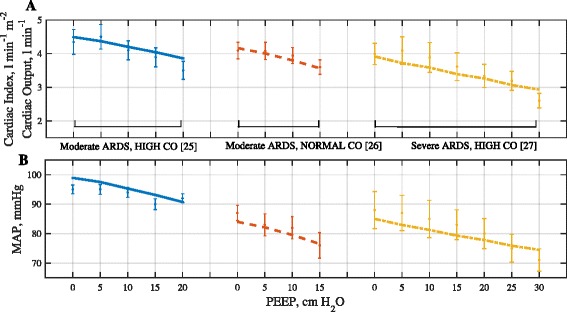
Results of fitting model outputs for hemodynamic variables to patient data. **a** Cardiac index (or Cardiac output for the case of Moderate ARDS, Normal CO) and **b** MAP. The lines represent the model results while the error bars depict the data. Three patients are: Moderate ARDS High CO (*blue*), Moderate ARDS Normal CO (*red*), Severe ARDS High CO (*yellow*)

The first *in silico* dataset consisted of three individual *in silico* ARDS patients that could be stratified by ARDS severity and different baseline cardiac output levels (Table 1). The first patient, data from [25], had a PF ratio of 150 mmHg and CO of 8 l min^−1^ (i.e. moderate severity ARDS, with high CO) at PEEP = 0 cm H_2_O. The second patient, data from [26], had a PF ratio of 167 mmHg and CO of 4.09 l min^−1^ (i.e. moderate severity ARDS, with normal CO), while the third patient, data from [27], had a PF ratio of 50 mmHg and CO of 7.3 l min^−1^ (severe ARDS, with high CO). This dataset was used to determine the model’s lung configuration to yield responses of PaO_2_ and PaCO_2_ corresponding to the static data values. Following this, the cardiovascular model parameters were configured to changes in CO and MAP at different values of PEEP (see Fig. 2).

**Fig. 3:**
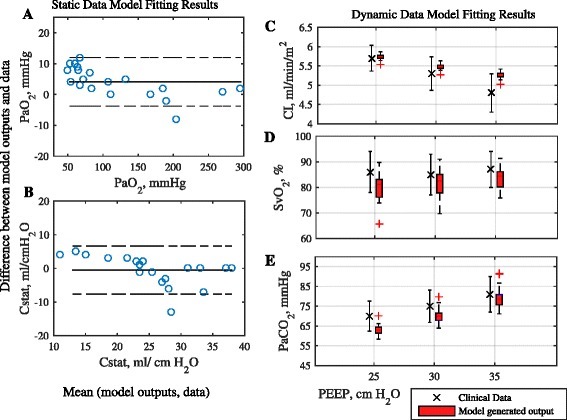
**a** and **b**: Bland Altman plots of difference in model outputs against values listed in data for PaO_2_ and Cstat respectively, plotted against mean of the model output and the data. Solid line represents bias and dashed lines represent 95% limits of agreement. Box plots in **c**, **d** and **e** depict the distribution of model generated values at different PEEP levels for Cardiac index (CI), mixed venous oxygen saturation (SvO_2_) and arterial carbon dioxide tension (PaCO_2_). The errorbars correspond to the population distribution of data at corresponding PEEP values

Table 2 lists the baseline characteristics of the second *in silico* dataset, comprising 20 patients with varying severity of ARDS, extracted from [5]. For each patient, the reported values of the ratio of P_a_O_2_ to fraction of oxygen in inhaled air (PF ratio) and the Cstat were used to fit lung configuration of the model at baseline settings of PEEP = 10 cm H_2_O and Pplat = 30 cm H_2_O (static data). The cardiovascular model parameters were then estimated to fit model responses to average values of CO, mixed venous oxygen saturation (SvO_2_) and PaCO_2_ at different PEEP levels (25, 30 and 35 cm H_2_O) (dynamic data) (see Fig. 3).

As stated above, the model was configured to reproduce data corresponding to ARDS patients in two stages, to static data at a single value of PEEP and then to dynamic data at varying values of PEEP. In the first stage, a global optimization algorithm was used to search for a configuration of lung parameters consisting of: threshold opening pressure (TOP), alveolar stiffness (*S*), extrinsic pressure (P_ext_) and microbronchial (inlet) resistance (R_alv_) for each alveolar compartment. Further objectives specified for the optimization were to keep average TOP to 20 cm H_2_O [28], and to keep Pplat below 30 cm H_2_O [29]. In the second stage of model matching, cardiovascular parameters in the model (e.g. compartmental elastances and blood volumes, arterial resistances, non-linear effects on pulmonary vascular resistance and intrathoracic ventricular splinting - see Additional file 1) were optimized to match observed changes in CO and MAP at different values of PEEP. All patients were assumed to have a weight of 70 kg and body surface area of 1.79 m^2^. Full details of how the model was matched to the patient data are provided in the Additional file 1.

### Recruitment maneuver protocols

**Fig. 4:**
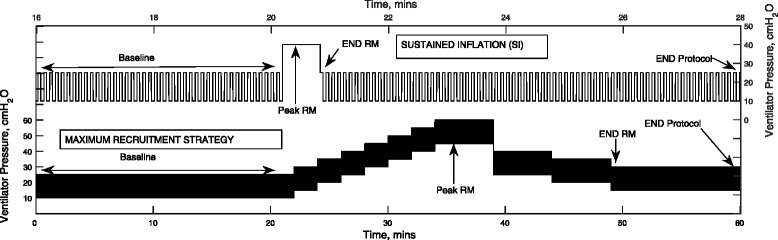
Ventilator pressure waveform for sustained inflation (SI) and maximal recruitment strategy (MRS)

RM protocols were executed by establishing a baseline steady-state condition for 20 min, executing the RM, and finally establishing a new post-RM steady-state. To establish the baseline condition, the simulated patients were subjected to identical PEEP (10 cm H_2_O) and identical inspiratory pressure (15 cm H_2_O above PEEP), as reported in [30]. Post RM, the inspiratory pressure is maintained at 15 cm H_2_O above PEEP. Throughout the protocols, only the ventilator pressure was altered. Two RMs from the published literature were implemented in the simulator, as detailed below and illustrated in Fig. 4.

### Maximal recruitment strategy (MRS) [30]

The maneuver comprises of PEEP adjustment in pressure-controlled mode, with a fixed driving pressure of 15 cm H_2_O (above PEEP). During the recruitment phase, PEEP was increased from 10 cm H_2_O to a maximum of 45 cm H_2_O in steps of 5 cm H_2_O, with each step lasting 2 min. During the PEEP-titration phase, the PEEP is set to 25 cm H_2_O and then reduced by 5 cmH_2_O in steps to the end-maneuver PEEP, with each step lasting 5 min. The PEEP titration was stopped when the percentage of recruited lung fell by more than 2% from maximal recruitment achieved during the recruitment phase. Although the ensuing higher airway pressures are a valid concern [31], studies have shown that the implementation of higher PEEP strategies with constant driving pressure does not lead to an increase in adverse outcomes [30, 32].

### Sustained inflation (SI) [2]

This was simulated as a sustained pulmonary inflation maneuver, with a positive ventilator pressure of 40 cm H_2_O applied for 40 s. The end-maneuver PEEP was set to 10 cmH_2_O.

## Results

### Model outputs accurately reproduce clinical datasets

The results of matching the model to data from [25–27] on 3 ARDS patients of varying ARDS severity and varying cardiac output are given in Table 1 and Fig. 2, and the results of the model matching to the dataset from [5] on 20 patients stratified by ARDS severity are shown in Table 2 and Fig. 3. All model outputs of interest are consistently very close to the values reported in the clinical data, confirming the ability of the simulator to reproduce physiological responses of individual patients.

### Evaluation of the maximal recruitment strategy (MRS) and Sustained inflation (SI) RMs on 3 *in silico* patients with varying ARDS severity and varying cardiac output

**Fig. 5:**
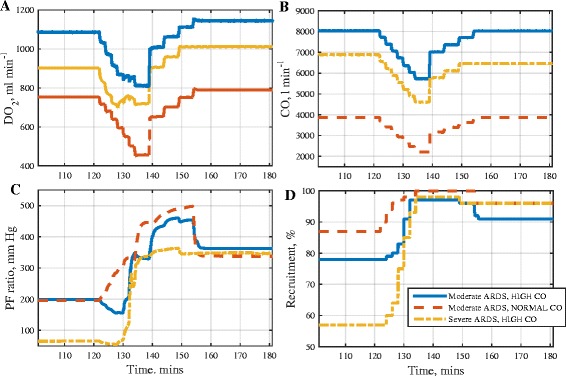
Results of applying the maximum recruitment strategy (MRS) to three *in silico* ARDS patients. Plots of: **a** oxygen delivery (DO_2_), **b** cardiac output (CO), **c** ratio of arterial oxygen tension to fraction of oxygen in inhaled air (PF ratio), **d** % of recruited lung (Recruitment)

Table 3 shows data on the results of executing the MRS on the 3 *in silico* ARDS patients from the first datset. Figure 5 shows time courses of oxygen delivery (DO_2_), CO, PF ratio and percentage of recruited lung. Time courses of right ventricle volume (V_RV_), physiological shunt (Shunt), PaCO_2_, MAP, MPAP, SaO_2_ and SvO_2_ are provided in the (Additional file 1: Figure S6). Key effects of the MRS maneuver can be summarized as follows:

**Table 3:** Key results of Recruitment Maneuvers in *in silico* ARDS patients with varying severity and cardiac output

	Moderate ARDS, High CO [25]		Moderate ARDS, Normal CO [26]		Severe ARDS, High CO [27]	
RM	MRS	SI	MRS	SI	MRS	SI
End RM PEEP, cm H_2_O	10	10	10	10	15	10
Recruitment_B_ (baseline), %	78	78	8	87	57	57
Recruitment_M_ (maximum), %	97	80	100	97	98	65.
R Ratio	19.59	2.50	13.00	10.31	41.84	12.31
∆ CO (at max P_AW_), l min^−1^	−2.3	−1.7	−1.6	−1.2	−2.3	−1.5
∆ RVEDV (at max P_AW_), ml	−14	−6	−55	−29	−18	−10
DO_2_ (baseline), ml min^−1^	1086	1086	754	754	902	902
∆ DO_2_ (at max P_AW_), ml min^−1^	810	834	453	540	697	689
∆ DO_2_ (post RM), ml min^−1^	1144	1097	790	786	1012	971
RAP (baseline), mm Hg	9	9	11	11	11	11
RAP (at max P_AW_), mm Hg	18	12	21	15	23	16
PF ratio (baseline), mm Hg	199	199	196	196	65	65
PF ratio (post RM), mm Hg	363	213	337	309	347	86

List of Abbreviations: *RM* recruitment maneuver, *PEEP* positive end expiratory pressure, *R Ratio* recruitment ratio ((recruitment_M_ -recruitment_B_)/recruitment_B_ × 100), ∆ *CO* change in cardiac output relative to baseline, ∆ *RVEDV* change in right ventricle end diastolic volume relative to baseline, *DO*_2_ oxygen delivery, ∆ *DO*_2_ change in oxygen deliver relative to baseline, *PF rat*io ratio of arterial partial pressure of oxygen to fraction of oxygen in inhaled air, *max P*_*AW*_ maximum airway pressure, *RAP* right atrial pressures

**Fig. 6:**
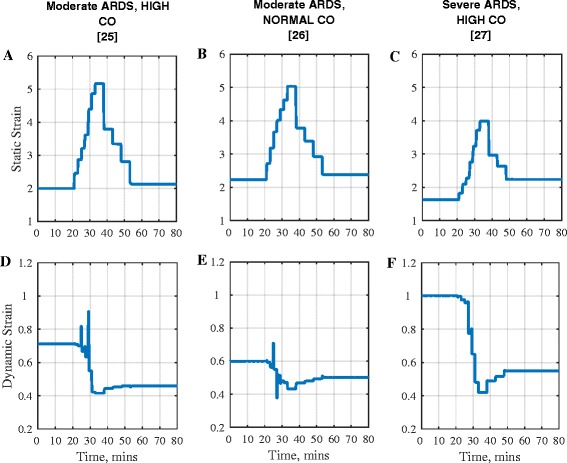
Strain in three *in silico *ARDS patient during MRS RM. Static strain in: **a** Moderate ARDS High CO, **b** Moderate ARDS Normal CO and **c** Severe ARDS High CO. Dyn﻿amic strain in **d** Moderate ARDS High CO, **e** Moderate ARDS Normal CO and **f** Severe ARDS High CO

- In all patients, large increases in PF ratio were observed during the application of the MRS, and PF ratio remained significantly greater than baseline values after the RM ended. Improved recruitment, reduced dynamic lung strain, and falls in arterial carbon dioxide levels were evident during and after the RM, indicating an increase in effective lung area and reduced ventilation/perfusion mismatch.
- DO_2_ fell by more than 200 ml min^−1^ in all three patients at maximum PEEP. This was caused by a decrease in CO which outweighed the increase in oxygen content in all cases, with the lowest CO occurring at maximum PEEP. In one patient (moderate ARDS, normal CO) the level of DO_2_ during the maneuver fell below 500 ml min^−1^, which would be likely to cause systemic responses, such as blood flow being redirected to critical organ systems, reducing tissue oxygenation in other tissue beds and potentially leading to residual organ dysfunction.
- The end-diastolic volume of the right ventricle fell as PEEP increased in all patients. The end-systolic volume remained relatively constant in the patients with high CO. Both CO and DO_2_ returned to close-to-baseline levels for the *in silico* patients with moderate ARDS as PEEP returned to 10 cm H_2_O.
- A significant post-RM increase in DO_2_ was maintained only in the *in silico* patient with severe ARDS.
- Figure 6 shows that in all *in silico* patients, the MRS led to an increase in static lung strain and a decrease in dynamic lung strain. The largest decrease in dynamic lung strain was observed in the *in silico* patient with severe ARDS.

**Fig. 7:**
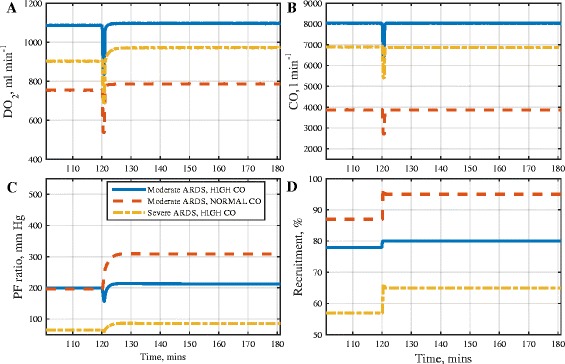
Results of applying the sustained inflation (SI) RM in three *in silico* ARDS patients. Plots of: **a** oxygen delivery (DO_2_), **b** cardiac output (CO), **c** ratio of arterial oxygen tension to fraction of oxygen in inhaled air (PF ratio),) **d ** % of recruited lung (Recruitment)

Table 3 also shows the results of executing the SI RM on the 3 *in silico* ARDS patients. Figure 7 shows time courses of DO_2_, CO, PF ratio and % of recruited lung. Time courses of other measured variables are provided in the (Additional file 1: Figure S7). Relative to the MRS, the hemodynamic changes during the SI RM lasted for a shorter duration, and its main effects can be summarized as follows:

- In the virtual patients with moderate severity ARDS, high CO and severe ARDS, high CO, small numbers of alveoli were re-opened, resulting in only small increases in PF ratio being attained.
- Significantly greater recruitment (and hence a larger increase in PF ratio) was observed in the moderate severity ARDS, normal CO subject. However, the resulting gain in oxygen content was effectively cancelled out by the reduction in cardiac output.
- Only small post-RM improvements in DO_2_ were observed in all three virtual patients, with the largest improvement being observed in the severe ARDS subject.

Due to the superior performance of the MRS with respect to PF ratio, recruitment, strain and post RM DO_2_, this RM was selected for further investigation using an additional dataset from a larger cohort of patients reported in [5].

### Evaluation of the maximal recruitment strategy RM on 20 *in silico* patients with varying ARDS severity and high cardiac output

Table 4 shows the results of executing the MRS on 20 in *silico* ARDS patients, with results listed for subsets of the patients, stratified based on the severity of ARDS. Key effects of the MRS for this *in silico* patient cohort can be summarized as follows:

**Table 4:** Key results of Maximum Recruitment Strategy (MRS) in 20 *in silico* ARDS patients

	All Patients		Severe ARDS		Moderate ARDS		Mild ARDS	
	mean	sd	mean	sd	mean	sd	mean	sd
PEEP (baseline), cmH_2_O	10.0	0	10.0	0	10.0	0	10.0	0
PEEP (post RM), cmH_2_O	24.5	2	25.0	0	25.0	0	21.7	6
Recruitment (baseline), %	63.3	18	49.7	11	75.7	4	88.0	11
Recruitment (at max P_AW_), %	93.7	3	92.4	2	94.2	3	97.7	3
CO (baseline), l min^−1^	11.6	0.2	11.7	0.1	11.6	0.2	11.3	0.3
CO (at max P_AW_), l min^−1^	8.3	0.3	8.4	0.2	8.2	0.2	8.1	0.3
CO (post RM), l min-1	10.3	0.2	10.3	0.1	10.2	0.1	10.4	0.1
∆ RVEDV (at max P_AW_), ml	−48	6.1	−49	6.2	−46	5.9	−52	4.6
∆ RVESV (at max P_AW_), ml	−33	21	−33	23	−43	16.2	−15	18
DO_2_ (baseline), ml min^−1^	1556	340	1316	244	1809	138	1929	194
DO_2_ (at max P_AW_), ml min^−1^	1398	81	1406	94	1385	74	1403	63
DO_2_ (post RM), ml min^−1^	1642	114	1595	99	1671	97	1759	124
P_RA_ (baseline), mmHg	6.5	4	6.7	4	5.5	4	7.8	6
P_RA_ (at max P_AW_), mmHg	7.8	4	8.8	4	7.3	4	4.9	0
P_RA_ (post RM), mmHg	7.6	4	7.4	4	6.4	4	10.7	0
PF ratio (baseline), mmHg	102	85	54	10	115	50	250	125
PF ratio (at max P_AW_), mmHg	206	72	182	53	207	81	298	48
PF ratio (post RM), mmHg	140	78	92	23	179	82	243	78
Dynamic strain (baseline),	0.18	0.03	0.18	0.04	0.19	0.03	0.18	0.02
Dynamic strain (at max P_AW_)	0.10	0.02	0.12	0..02	0.10	0.02	0.09	0.01

List of Abbreviations: *RM* recruitment maneuver, *PEEP* positive end expiratory pressure, *CO* cardiac output, ∆ *RVEDV* change in right ventricle end diastolic volume relative to baseline, ∆ *LVEDV* change in left ventricle end diastolic volume relative to baseline, ∆ *RVESV* change in right ventricle end systolic volume relative to baseline, ∆ *LVEDV* change in left ventricle end systolic volume relative to baseline, *P*_*RA*_ right atrial pressure, *DO*_2_ oxygen delivery, *PF ratio* ratio of arterial partial pressure of oxygen to fraction of oxygen in inhaled air, *max P*_*AW*_ maximum airway pressure

- PF ratio increased on average by 105 mmHg during the application of the MRS, and remained significantly greater than baseline values afterwards. The biggest improvement in PF ratio was seen in the severe ARDS subgroup. Improved recruitment and reduced dynamic strain were evident for all *in silico* patients during the MRS.
- DO_2_ fell by more than 150 ml min^−1^ on average during application of the MRS. This fall in DO_2_ occurred mostly in the *in silico* patients with moderate and mild ARDS, whereas in the severe patients DO_2_ increased by nearly 90 ml min^−1^ on average during the RM. Due to the high baseline CO of the patients in this cohort, DO_2_ remained above 1000 ml min^−1^ in all *in silico* patients at all times, indicating no risk of tissue de-oxygenation due to application of the RM.
- The end-diastolic and end-systolic volume of the right ventricle fell as PEEP increased. The decrease in end-systolic volume was smaller than the decrease in end-diastolic volume, indicating a smaller stroke volume at maximum PEEP. There was a small increase in the right atrial pressure as PEEP was increased.

## Discussion

Oxygen is essential for cellular metabolism and delivery of sufficient levels of oxygen is vital to preserve organ function. Accordingly, early correction of tissue hypoxia is an important task in management of critically ill patients in intensive care units. DO_2_ is a well-known and relatively simple surrogate estimate for the oxygen delivered to the cells from the lungs, determined by CO and arterial oxygen content. Although some early studies suggested that there were beneficial outcomes associated with increasing DO_2_ levels in certain population [33–35], aggressive DO_2_ targeted protocols were found to be ineffective and potentially harmful in major randomized controlled studies [36, 37]. This was attributed to extreme fluid loading and excessive use of vasoactive agents [36, 38]. High PEEP recruitment maneuvers such as the MRS have shown the potential to increase arterial oxygen content through recruitment of collapsed regions of the lung in ARDS patients, both in clinical trials [30] and in computational studies [16]. This raises the question of whether periodic RMs could be used to improve the delivery of oxygen without the need for aggressive fluid loading, whilst minimizing the continual stress effect of high intrathoracic pressure on the cardiovascular system.

The results of this study indicate that in *in silico* patients with mild or moderate ARDS, the reduction in cardiac output caused by the RMs (Table 4) could potentially prevent any significant improvements in oxygen delivery that might be expected due to improved gas exchange; this finding is consistent with some previous clinical studies [8, 39]. This phenomenon was more pronounced in those subjects with less severe hypoxia (who therefore had a smaller number of recruitable alveoli), leading to little improvement in DO_2_ post-RM. This trade-off (which was seen in a substantial patient group) may partly explain the lack of demonstrated outcome benefit seen to date when RMs are applied to non-stratified ARDS patients [40].

In the *in silico* patients with severe ARDS, with more alveoli available for recruitment, a larger improvement in DO_2_ was evident after the application of the MRS (Table 4 and Fig. 5a). In fact in the second dataset of 20 *in silico* patients, an increase in DO_2_ was observed in those patients with severe ARDS even as PEEP was incremented and CO was falling during the recruitment phase of the RM. This response was also observed in the severe ARDS *in silico* patient from the first dataset. In this case, DO_2_ did not fall in tandem with CO during the whole duration of the RM. Between the time interval of 25 and 30 min (Fig. 5a), DO_2_ actually increased (even while CO continued to fall). The mechanism of rise in DO_2_ in these cases can be attributed to the substantial increase in alveolar recruitment, enhancing arterial oxygenation. The SI RM, in contrast, produced significantly less recruitment. Thus, the results from our *in silico* trials suggest that those patients with the most severe acute lung injury may benefit most from high-PEEP recruitment strategies, as also suggested in [41].

Aside from improving oxygenation, another goal of RMs is to reduce the risk of atelectrauma. The strain plots of Fig. 6 and results from Table 3 show that in the *in silico* patients with higher lung recruitability, higher PEEP and the consequent reduction in tidal opening and closing of alveolar compartments helps in improving dynamic strain. This agrees with data from previous animal studies [24]. This is accompanied by increased static strain as a result of higher end expiratory volumes. Studies have suggested that large static strain may be better tolerated than equivalent dynamic strain [24, 42] and may be beneficial, due to a more homogenous lung ventilation [43].

Several studies have found associations between maintaining sufficient oxygen delivery and positive patient outcomes. For example, it was reported in [35] that maintaining DO_2_ above 600 ml min^−1^ m^2^ was associated with reduced post-surgery complications and shorter hospital stays in post-surgery patients, while DO_2_ levels of less than 10.9 ml min^−1^ kg^−1^ at cardiac index = 3.1 L min^−1^ m^2^ were associated with a higher risk of mortality [44]. The potential for substantial negative influence of positive intrathoracic pressure on oxygen delivery is most clearly exhibited in the *in silico* patient with moderate severity ARDS and normal cardiac output from the first dataset (Fig. 5b). During both RMs, improvements in oxygenation occurred in tandem with large decreases in CO, resulting in DO_2_ levels falling to values that could potentially lead to organ dysfunction. Neither RM produced a significant long-term improvement in oxygen delivery.

Our results indicate that a higher initial cardiac output may confer relative protection from reductions in stroke volume due to high intrathoracic pressures occurring during RMs. This, however, might not be entirely reflective of all cases of acute cor pulmonale associated with severe ARDS, which can occur in up to a third of these patients [45]. In those instances, volume overload can actually have deleterious effects. We plan to investigate the effects of severe ARDS on the right ventricle as another aspect of heart-lung interactions in subsequent investigations.

The patients in the second dataset have significantly higher baseline values of CO and DO_2_. These values are consistent with the data in [5], which reported a mean cardiac index of 5.8 l min^−1^m^−2^ at Pplat of 30 cmH_2_O. The relationship between RM based increases in intrathoracic pressure and depression of cardiac output may also be more complicated than suggested by the relatively simplistic initial cardiovascular state stratification of high/low CO presented here. For example, the effect of respiratory variation on inferior vena cava diameter or RAP can result in PEEP induced decreased venous return and cardiac output [10]. In this case, an ARDS patient with sepsis as the trigger, reduced afterload and appropriately managed with a conservative fluid strategy could have a high cardiac output but still be expected to be fluid responsive and have a significant drop in cardiac output during a RM. Yet this would not be seen in a patient with sepsis associated cardiomyopathy with low/normal cardiac output operating on the flat portion of their RV Frank-Starling function curve. Our ability to draw conclusions about the precise presence or absence of cardiopulmonary dysfunction is limited from what is information is available in published data sets. However, we note that cyclical cardiovascular changes in venous, ventricular and arterial systems in response to periodic intrathoracic pressure from ventilation are observable in the model. This signal change is consistent with the dynamic indices of fluid responsiveness in response to tidal ventilation, and we plan to investigate this further during simulated hemorrhage and re-transfusion to help to further calibrate and validate the cardiovascular aspects of our integrated model.

The simulation model used in this study has some limitations. The autonomic reflexes are neglected because, in the studies used for model calibration [5, 25–27], it is likely that the cardiovascular side effects of the drugs and dosages used for sedation suppressed normal cardiovascular system baroreceptor reflexes (these studies consistently reported no significant changes in heart rate throughout their interventions). Effects due to increased cytokine presence in the systemic circulation due to alveolar-capillary membrane damage are not included. Their precise role in terms of isolated systemic effects on the vasculature is difficult to quantify in a clinical setting, since ethical considerations would require some amount of treatment to reverse the adverse effects associated with these changes, such as drugs to improve hypotension. However, we do measure and quantify alveolar strain, which has been established as a reliable surrogate for lung damage that exacerbates barotrauma [24].

Finally, previous studies have shown that the systemic pressure can compensate in response to changes in PEEP, attributed to neuromuscular reflexes [10, 46] and alveolar recruitment can lead to simultaneous recruitment of pulmonary vessels, increasing the vascular volume, and reducing the pulmonary artery pressure [47]. These mechanisms were omitted from the model due to a lack of reliable data for model calibration, and because the drugs and dosages used to produce the type of ventilation seen in the studies on which our model has been calibrated strongly suggest complete muscle relaxation and a constant total body VO_2_.

## Conclusions

An integrated cardiopulmonary computational model was shown to be able to accurately match the cardiorespiratory responses of 23 individual patients with varying severity of ARDS and CO levels. The resulting bank of *in silico* patients allowed us to perform an in-depth and controlled investigation of the effect of lung recruitment maneuvers on key patient outcome parameters. Our results support the hypothesis that patients with severe ARDS (and hence worse starting VQ mismatch and more alveolar units available for recruitment) may benefit more from RMs. Our results also indicate that a higher than normal initial cardiac output may provide protection against the effects of high intrathoracic pressures associated with RMs on cardiac function. Results from the *in silico* patients with mild or moderate ARDS suggest that the detrimental effects of RMs on cardiac output can potentially outweigh the positive effects on oxygenation through alveolar recruitment, resulting in overall reductions in tissue oxygen delivery. However, RMs have other potential benefits aside from improved oxygenation, e.g. reduction in atelectrauma. In such patient groups, it may therefore still be useful to administer RMs as long as no dangerous decline in cardiac function is observed. Clinical trials using stratified patient groups could confirm the results of this *in silico* study and allow the development of more effective guidelines for the application of RMs in ARDS treatment.

## Additional file

Additional file 1: Model and Model fitting description and calibration. Contains description of the pulmonary model, cardiac model, cardio pulmonary interactions, model calibration to a healthy state and disease state, selection of patient data, assignment of baseline model parameters, model parameter configuration using optimization, list of parameters used for model fitting, model parameters for simulated patients and healthy state, hemodynamic and pulmonary outputs. (PDF 1930 kb)

## Abbreviations

ARDS: Acute respiratory distress syndrome
CI: Cardiac index [ml min^−1^ m^2^]
CO: Cardiac output [ml min^−1^]
CO_2_: Carbon dioxide
DO_2_: Oxygen delivery [ml min^−1^]
F_I_O_2_: Fraction of inspired air constituting of oxygen.
Hb: Haemoglobin content in blood [gm l^−1^]
IE: Inspiratory to expiratory ratio
LV: Left ventricle
MAP: Mean arterial pressure [mm Hg]
MPAP: Mean pulmonary arterial pressure [mm Hg]
MRS: Maximum recruitment strategy
O_2_: Oxygen
PaO_2_: Partial pressure of oxygen in arterial compartment [kpa]
PEEP: Positive end expiratory pressure [cm H_2_O]
P_ext_: Extrinsic pressure attributed to an alveolar unit in the model [cm H_2_O]
PF ratio: Ratio of arterial pressure of oxygen to fraction of oxygen in inspired air
pH_a_: Arterial pH value
P_IT_: Intrathoracic pressures
Pplat: Plateau pressure [cm H_2_O]
PvO_2_: Partial pressure of mixed venous oxygen [kpa]
R_alv_: Microbronchial (inlet) resistance attributed to an alveolar unit in the model
RM: Recruitment maneuver
RQ: Respiratory quotient
RV: Right ventricle
RVEDV: Right ventricle end diastolic volume [ml]
RVESV: Right ventricle end systolic volume [ml]
S_alv_: Alveolar stiffness, attributed to an alveolar unit in the model
SaO_2_: Arterial oxygen saturation
SI: Sustained inflation
SvO_2_: Venous oxygen saturation
TOP: Threshold opening pressure [cm H_2_O]
TOPalv: Threshold opening pressure attributed to an alveolar unit in the model [cm H_2_O]
VILI: Ventilator induced lung injury
VO_2_: Oxygen consumption [ml min^−1^]
VQ: Ventilation perfusion
VR: Respiration rate set by the ventilator [breaths per minute, bpm]
