# Supplementary material for: Hemodynamic effects of lung recruitment maneuvers in acute respiratory distress syndrome
Source: BMC Pulm Med. 2017 Feb 8;17:34. doi: 10.1186/s12890-017-0369-7 (PMC5299789; doi:10.1186/s12890-017-0369-7)
Supplement: Additional file 1: — Model and Model fitting description and calibration. Contains description of the pulmonary model, cardiac model, cardio pulmonary interactions, model calibration to a healthy state and disease state, selection of patient data, assignment of baseline model parameters, model parameter configuration using optimization, list of parameters used for model fitting, model parameters for simulated patients and healthy state, hemodynamic and pulmonary outputs. (PDF 1930 kb) [file 12890_2017_369_MOESM1_ESM.pdf]

Hemodynamic effects of lung recruitment maneuvers in acute respiratory distress syndrome

Model description and calibration

Anup Das[1], Marc Chikhani[2], Oana Cole[2], Wenfei Wang[1], Tayyba Ali[2], Mainul Haque[2], Declan G. Bates[1] and Jonathan G. Hardman[2]

[1]School of Engineering, University of Warwick, CV4 7AL, UK

[2]School of Medicine, University of Nottingham, NG7 2UH, UK

Corresponding Author: Prof. Declan G. Bates, School of Engineering, University of Warwick, CV4 7AL, UK, Email: D.Bates@warwick.ac.uk

Additional File

The online data supplement for this paper contains additional material that could not be included in the main text due to space limitations, and is divided into four sections. The following document describes in detail the simulation model employed in the paper. The optimization strategy used in fitting the model to the ARDS patient data is also described.

Contents

Section 1 - Description of the pulmonary model 3

Section 2 - Description of the Cardiac model 10

Section 3 - Cardio pulmonary interactions 12

Section 4 - Model calibration to a healthy state 13

Section 5 - Model calibration to disease state 15

- Selection of Patient Data 15
- Assignment of baseline model parameters 15
- Model parameter configuration using optimization 16

Section 6 - List of parameters used for model fitting 18

Section 7 - Model parameters for simulated patients and healthy state 19

Section 8 – Other key hemodynamic and pulmonary parameters 20

References..22

Section 1 - Description of the pulmonary model

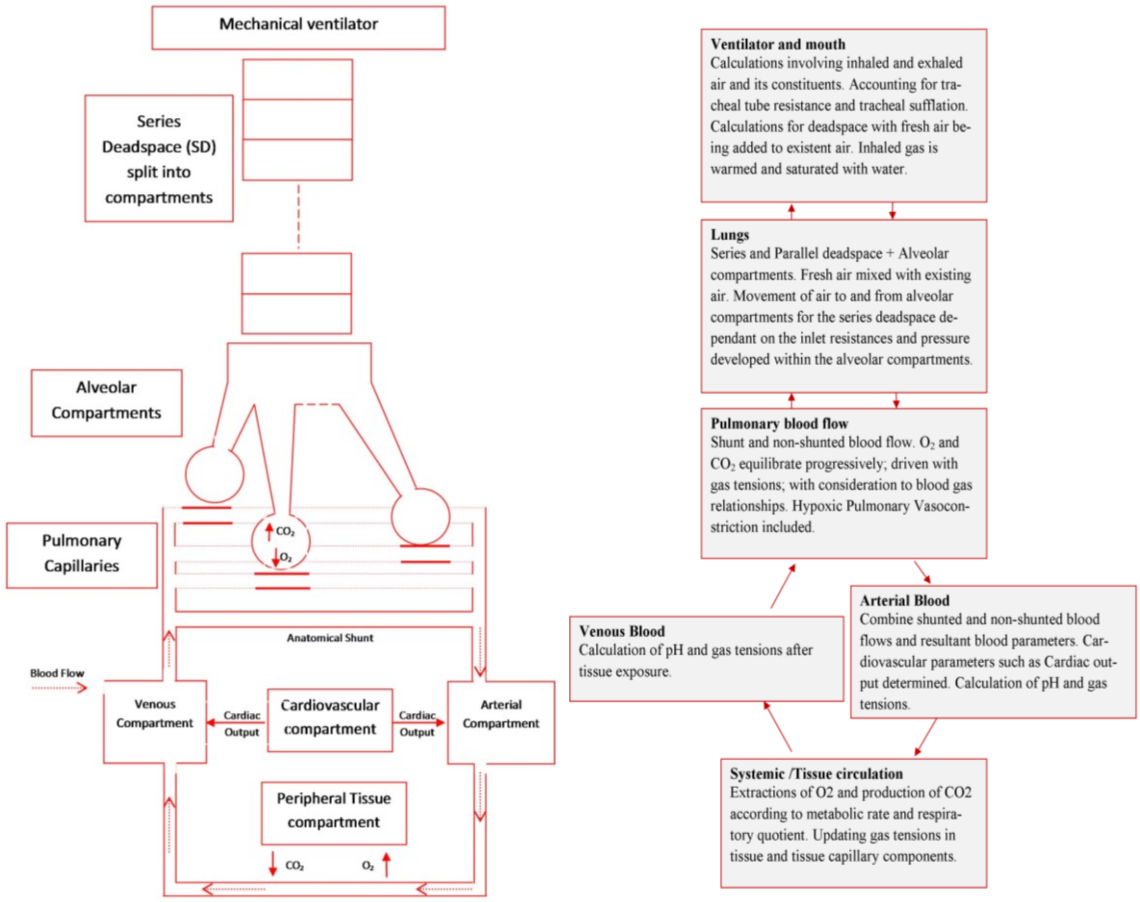

FigureS1: Diagrammatic representation of the model and its main features.

The model employed in this paper has been developed over the past several years and has been applied and validated on a number of different studies [1-8]. The model is organized as a system of several components, each component representing different sections of pulmonary dynamics and blood gas transport, e.g. the transport of air in the mouth, the tidal flow in the airways, the gas exchange in the alveolar compartments and their corresponding capillary compartment, the flow of blood in the arteries, the veins, the cardiovascular compartment, and the gas exchange process in the peripheral tissue compartments. Each component is described as several mass conserving functions and solved as algebraic equations, obtained or approximated from the published literature, experimental data and clinical observations. These equations are solved in series in an iterative manner, so that solving one equation at current time instant $(\mathrm{t_k})$ determines the values of the independent variables in the next equation. At the end of the iteration, the results of the solution of the final equations determine the independent variables of the first equation for the next iteration.

The iterative process continues for a predetermined time, *T*, representing the total simulation time, with each iteration representing a 'time slice' *t* of real physiological time (set to 30 ms). At the first iteration $(\mathrm{t_k}, \mathrm{k} = 0)$, an initial set of independent variables are chosen based on values selected by the user. The user can alter these initial variables to investigate the response of the model or to

simulate different pathophysiological conditions. Subsequent iterations ($\mathrm{t_k} = \mathrm{t_{k-1}} + t$) update the model parameters based on the equations below.

The pulmonary model consists of the mechanical ventilation equipment, anatomical and alveolar deadspace, anatomical and alveolar shunts, ventilated alveolar compartments and corresponding perfused capillary compartments. The pressure differential created by the mechanical ventilator drives the flow of gas through the system. The series deadspace (SD) is located between the mouth and the alveolar compartments and consists of the trachea, bronchi and the bronchioles where no gas exchange occurs. Inhaled gases pass through the SD during inspiration and alveolar gases pass through the SD during expiration. In the model, an SD of volume 60ml is split into 50 stacked layers of equal volumes (N_{SD} = 50). No mixing between the compartments of the SD is assumed.

Any residual alveolar air in the SD at the end of expiration is re-inhaled as inspiration is initiated. This residual air is composed of gases exhaled from both perfused alveolar compartments (normal perfusion) and the parallel deadspace (PD) (alveolar compartments with limited perfusion). Therefore, the size of deadspace (SD and PD) can have a significant effect on the gas composition of the alveolar compartments.

The inhaled air is initially assumed to consist of five gases: oxygen (O_2), nitrogen (N_2), carbon dioxide (CO_2), water vapour (H_2O) and a 5th gas (α) used to model additives such as helium or other anaesthetic gases. During an iteration of the model, the flow (*f*) of air to or from an alveolar compartment *i* at time $\mathrm{t_k}$ is determined by the following equation:

$$f_i(\mathrm{t_k}) = \frac{(p_v(\mathrm{t_k}) - p_i(\mathrm{t_k}))}{(\mathrm{R_u} + \mathrm{R}_{\mathrm{A},\,i})} \qquad \text{for } i = 1, \dots, N_A \tag{1}$$

where $p_v(\mathrm{t_k})$ is the pressure supplied by the mechanical ventilator at $(\mathrm{t_k})$, $p_i(\mathrm{t_k})$ is the pressure in the alveolar compartment i at $(\mathrm{t_k})$, $\mathrm{R_u}$ is the constant upper airway resistance and $\mathrm{R}_{\mathrm{A},\,i}$ is the bronchial inlet resistances of the alveolar compartment i. N_A is the total number of alveolar compartments (for the results in this paper, N_A = 100). The total flow of air entering the SD at time $\mathrm{t_k}$ is calculated by

$$f_{SD}(\mathrm{t_k}) = \sum_{i=1}^{N_A} f_i(\mathrm{t_k}) \tag{2}$$

During the inhaling phase, $f_{SD} \geq 0$, while in the exhaling phase $f_{SD} < 0$.

During gas movement in the SD, the fractions of gases in the layer l of the SD, $\mathrm{F}_{l,}$ $(l = 1, \dots, N_{SD})$ is updated based on the composition of the total flow, f_{SD}, and the current composition of $\mathrm{F}_{l,}$. If $f_{SD} \geq$ 0, then air starts filling from the top layer (l = 1) to the bottom layer ($l = N_{SD}$); and vice versa for f_{SD} < 0.

The volume of gas x, in the ith alveolar compartment ($\mathrm{v}_{i,\,\mathrm{x}}$), is given by:

$$v_{i,\mathrm{x}}(\mathrm{t_k}) = \begin{cases} v_{i,\mathrm{x}}(\mathrm{t_{k-1}}) - f_i(\mathrm{t_k}) \cdot \frac{v_{i,\mathrm{x}}(\mathrm{t_{k-1}})}{v_i(\mathrm{t_k})} & \textit{Exhaling} \\ v_{i,\mathrm{x}}(\mathrm{t_{k-1}}) + f_i(\mathrm{t_k}) \cdot \mathrm{F}_{N_{SD}}(\mathrm{t_k}) & \textit{Inhaling} \end{cases} \qquad \text{for } i = 1, \dots, N_A \tag{3}$$

In (3), x is any of the five gases (O_2, N_2, CO_2, H_2O or α). The total volume of the ith alveolar compartment, v_i is the sum of the volume of the five gases in the compartment.

$$v_i(t_k) = v_{i,O2}(t_k) + v_{i,N2}(t_k) + v_{i,CO2}(t_k) + v_{i,H2O}(t_k) + v_{i,\alpha}(t_k) \tag{4}$$

For the alveolar compartments, the tension at the centre of the alveolus and at the alveolar capillary border is assumed to be equal. The respiratory system has an intrinsic response to low oxygen levels in blood which is to restrict the blood flow in the pulmonary blood vessels, known as Hypoxic Pulmonary Vasoconstriction (HPV). This is modelled as a simple function, resembling the stimulus response curve suggested by Marshall [9], and is incorporated into the simulator to gradually constrict the blood vessels as a response to low alveolar oxygen tension. The atmospheric pressure is fixed at 101.3kPa and the body temperature is fixed at 37.2°C.

At each t_k, equilibration between the alveolar compartment and the corresponding capillary compartment is achieved iteratively by moving small volumes of each gas between the compartments until the partial pressures of these gases differ by <1% across the alveolar-capillary boundary. The process includes the nonlinear movement of O_2 and CO_2 across the alveolar capillary membrane during equilibration.

In blood, the total O_2 content (C_{O2}) is carried in two forms, as a solution and as oxyhaemoglobin (saturated haemoglobin):

$$C_{O2}(t_k) = S_{O2}(t_{k-1}) \cdot Huf \cdot Hb + P_{O2}(t_{k-1}) \cdot O_{2sol} \tag{5}$$

In this equation, S_{O2} is the hemoglobin saturation, *Huf* is the Hufner constant, Hb is the hemoglobin content and O_{2sol} is the O_2 solubility constant. The following pressure-saturation relation, as suggested by [10] to describe the O_2 dissociation curve, is used in this model:

$$S_{O2}(t_k) = \left(\left(\left(P_{O2}^3(t_{k-1}) + 150 \cdot P_{O2}(t_{k-1})\right)^{-1} \times 23400\right) + 1\right)^{-1} \tag{6}$$

S_{O2} is the saturation of the hemoglobin in blood and P_{O2} is the partial pressure of oxygen in the blood. As suggested by [11], P_{O2} has been determined with appropriate correction factors in base excess BE, temperature T and pH (7.5005168 = pressure conversion factor from kPa to mm Hg):

$$P_{O2}(t_k) = 7.5006168 \cdot P_{O2}(t_{k-1}) \cdot 10^{[0.48(pH(t_{k-1})-7.4) - 0.024(T-37) - 0.0013 \cdot BE]} \tag{7}$$

The CO_2 content of the blood (C_{CO2}) is deduced from the plasma CO_2 content ($C_{CO2plasma}$) [12] by the following equation:

$$C_{CO2}(t_k) = C_{CO2plasma}(t_{k-1}) \cdot \left[1 - \frac{0.0289 \cdot Hb}{(3.352 - 0.456 \, .S_{O2}(t_k)) \cdot (8.142 - pH(t_{k-1}))}\right] \tag{8}$$

where S_{O2} is the O_2 saturation, Hb is the hemoglobin concentration and pH is the blood pH level. The coefficients were determined as a standardized solution to the McHardy version of Visser's equation [13], by iteratively finding the best fit values to a given set of clinical data. The value of $C_{CO2plasma}$ is deduced using the Henderson-Hasselbach logarithmic equation for plasma C_{CO2} [14] :

$$C_{CO2plasma}(t_k) = 2.226 \cdot s_{CO2} \cdot P_{CO2}(t_{k-1}) \left(1 + 10^{(pH(t_{k-1}) - pK')}\right) \tag{9}$$

where s_{CO2} is the plasma CO_2 solubility coefficient and pK' is the apparent pK (acid dissociation constant of the CO_2 bicarbonate relationship). P_{CO2} is the partial pressure of CO_2 in plasma and

‘2.226’ refers to the conversion factor from miliMoles per liter to ml/100ml. [14] gives the equations for s_{CO2} and pK' as:

$$s_{CO2} = 0.0307 + 0.0057 \cdot (37 - T) + 0.00002 \cdot (37 - T)^2 \quad (10)$$

$$pK' = 6.086 + 0.042 \cdot (7.4 - pH(t_{k-1})) + (38 - T) \cdot \left(0.00472 + \left(0.00139 - (7.4 - pH(t_{k-1}))\right)\right) \quad (11)$$

$P_{CO2}(t_k)$ is determined by incorporating the standard Henry’s law and the s_{CO2} (the CO_2 solubility coefficient above). For pH calculation, the Henderson Hasselbach and the Van Slyke equation [15] are combined. Below is the derivation of the relevant equation. The Henderson-Hasselbach equation (governed by the mass action equation (acid dissociation)) states that:

$$pH = pK + \log\left(\frac{bicarbonate\ concentration}{carbonic\ acid\ concentration}\right) \quad (12)$$

Substituting pK=6.1 (under normal conditions) and the denominator $(0.225 \cdot P_{CO2})$ (acid concentration being a function of CO_2 solubility constant 0.225 and P_{CO2} (in kPa)) gives:

$$pH(t_k) = 6.1 + \log\left(\frac{HCO_3(t_{k-1})}{0.225 \cdot P_{CO2(t_k)}}\right) \quad (13)$$

For a given pH, base excess (BE), and hemoglobin content (Hb), HCO_3 is calculated using the Van-Slyke equation, as given by [15]:

$$HCO_3(t_k) = \left((2.3 \times Hb + 7.7) \times (pH(t_k) - 7.4)\right) + \frac{BE}{(1 - 0.023 \times Hb)} + 24.4 \quad (14)$$

The capillary blood is mixed with arterial blood using the equation below which considers the anatomical shunt (Sh) with the venous blood content of gas x ($C_{v,x}$), the non-shunted blood content from the pulmonary capillaries ($C_{cap,x}$), arterial blood content ($C_{a,x}$), the arterial volume (v_a) and the cardiac output (CO).

$$C_{a,x}(t_k) = \frac{CO(t_k) \cdot \left(Sh \cdot C_{v,x}(t_k) + (1 - Sh) \cdot C_{cap,x}(t_k)\right) + C_{a,x}(t_k) \cdot (v_a(t_k) - CO(t_k))}{v_a(t_k)} \quad (15)$$

The peripheral tissue model consists of a single tissue compartment, acting between the peripheral capillary and the *active* tissue (undergoing respiration to produce energy). The consumed O_2 (V_{O2}) is removed and the produced CO_2 (V_{CO2}) is added to this tissue compartment. Similarly to alveolar equilibration, peripheral capillary gas partial pressures reach equilibrium with the tissue compartment partial pressures, with respect to the nonlinear movement of O_2 and CO_2. Metabolic production of acids, other than carbonic acid via CO_2 production, is not modeled. After peripheral tissue equilibration of gases, the venous calculations of partial pressures, concentrations and pH calculations are done using comparable equations as above.

A simple equation of renal compensation for acid base disturbance is incorporated. The base excess (BE) of blood under normal conditions is zero. BE increases by 0.1 per time slice if pH falls below 7.36 (to compensate for acidosis) and decreases by 0.1 per time slice if pH rises above 7.4 (under alkalosis).

The simulated patient is assumed to be under complete mechanical ventilation. Consequently, the effects of ventilatory autoregulation by the patient have not been incorporated into the models.

Each alveolar compartment has a unique and configurable alveolar compliance, alveolar inlet resistance, vascular resistance, extrinsic (interstitial) pressure and threshold opening pressure. For the ith compartment of *N* alveolar compartments, the pressure p_i is determined by:

$$p_i(\mathrm{t_k}) = \begin{cases} S_i(v_i(\mathrm{t_k}) - V_c)^2 - P_{ext,i} & v_i(\mathrm{t_k}) > 0 \\ 0 & \end{cases} \quad \text{for } i = 1, \dots, N_A \tag{18}$$

where

$$S_i = k_i {N_A}^2/200000 \quad \text{and} \quad V_c = 0.2V_{FRC}/N_A$$

Equation (18) determines the alveolar pressure p_i (as the pressure above atmospheric in cm H_2O) for the ith compartment of *N* number of alveolar compartments for the given volume of alveolar compartment, $v_i(t)$ in milliliters. The alveolar compartments are arranged in parallel and interact with the series deadspace with respect to the movement of gases. The flow of air into the alveolar compartments is achieved by a positive pressure provided by the ventilator and the air moves along the pressure gradient. The equation models the behavior of the intact lung / chest-wall complex. The use of the square of the difference between v_i and *Vc* causes alveolar pressure to increase at volumes below *Vc*, leading to exhalation and a tendency to "snap shut" (mathematical note: the pressure with respect to volume is thus a U-shaped curve)[16].

P_{ext} (per alveolar unit, in cm H_2O) represents the *effective net pressure* generated by the sum of the effects of factors *outside each alveolus* that act to distend that alveolus; positive components include the outward pull of the chest wall, and negative effects include the compressive effect of interstitial fluid in the alveolar wall. Incorporating P_{ext} in the model allows us to replicate the situation of alveolar units that have less structural support or that have interstitial oedema, and thus have a greater tendency to collapse. A negative value of P_{ext} indicates a scenario where there is compression from outside the alveolus causing collapse. The parameter S_i is a scalar that determines the intra-alveolar pressure for a given volume (with respect to a constant collapsing volume V_c) and is dependent on the parameter k. The units of S_i are cm H_2O ml^{-2}. Finally, V_c is defined as a "constant collapsing volume" at which the alveolus tends to empty (through Laplace effects) and represents a fundamental mechanical property of tissue and surfactant [16]. V_{FRC} is the resting volume of the lung (assumed to be 3 litres).

The effect of the three parameters on the volume–pressure relationship of the alveolar compartments can be observed in the following Figure S2.

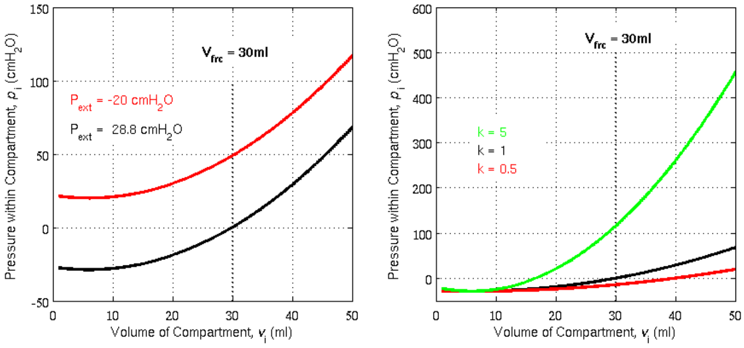

Figure S2: The effect of varying the parameters of Equation (18) on the pressure volume relationship of the model.

For a healthy lung at the end of the expiration, the ventilator pressure would return to zero above atmospheric (resulting in the tracheal pressure also being equal to zero). The nominal values for ($P_{ext,i}$, S_i) have been determined such that at the end of expiration, the alveolar pressure within the compartment is also equal to zero, i.e. at 30 ml, the individual compartments are at rest and consequently the total resting volume of the lung is 3 liters.

We consider each of the three parameters mentioned above ($P_{ext,i}$, S_i) to be different yet essential components for representing a diseased lung, that affect the volume pressure relationship of the alveolar compartments. For example, for a given volume v_i, increasing S_i increases the corresponding alveolar pressure of the alveolar compartment. When compared to another compartment with a lower S_i, a larger pressure from the mechanical ventilator would be needed to drive air into the compartment; thus effectively the compartment will be behaving as a stiffer lung unit.

Decreasing $P_{ext,i}$ increases the alveolar pressure such that the pressure gradient (especially during exhaling) forces the air out of the alveolar compartment until the volume of the compartment collapses (v_i = 0 ml). Note that, in effect, the parameters are influencing the resting volume of the compartments (when the alveolar pressure, p_i, is equal to zero). If p_i< 0 cm H_2O, the pressure gradient will cause the flow into the alveolar compartment (as ventilator pressure will always be ≥ 0 cm H_2O) until p_i reaches 0 cm H_2O.

In the model, the airway resistance $R_{aw}$is determined by the following equation for N parallel compartments:

$$\frac{1}{R_{aw}} = \frac{1}{R_{B,1}} + \frac{1}{R_{B,2}} + \cdots + \frac{1}{R_{B,N_A}}, \text{for } i = 1, \ldots, N_A \quad (19)$$

where $R_{B,i}$ is the bronchial inlet resistance of the ith compartment, which is defined by:

$R_{B,i} = m_i R_{B0}$

where R_{B0} corresponds to the default bronchial inlet resistance of an alveolar compartment. R_{B0} is set to $1 \times 10^{-5} \cdot \mathrm{N}$ (the inlet resistance is higher for a model with more compartments as the volume of each compartment decreases) for a healthy lung, giving a resistance of 0.001 kpa per ml per minute for 100 compartments. m_i is a coefficient of the airway resistance, representing a dynamic change in airway resistance and is determined by the equation:

$$m_i = \begin{cases} 1, & \mathrm{t}_{\mathrm{o},i} \le 0 \\ 10^{10}, & \mathrm{t}_{\mathrm{o},i} > 0 \end{cases} \quad \text{for } i = 1, \ldots, N_A \quad (20)$$

where,

$$\mathrm{t}_{\mathrm{o},i} = \begin{cases} \mathrm{t}_{\mathrm{o},i} - t\,, & p_{trachea} \ge \mathrm{TOP}_i \\ \tau_{c,i}, & p_{trachea} < \mathrm{TOP}_i \end{cases} \quad \text{for } i = 1, \ldots, N_A \quad (21)$$

$p_{trachea}$ is the pressure in the trachea and TOP_i is a value between 5 and 50 cm H_2O for the ith alveolar compartment. Additionally, a threshold opening pressure (TOP) at low lung volumes needs to be attained for a collapsed alveolar unit to open. Recruitment is a time dependent process, with different airways recruiting at different times, once the threshold pressure has been achieved [17, 18]. The equations within the model are solved iteratively as a discretized system. Each iteration represents a physiological time slice of t (10 ms). The time dependant recruitment phenomenon is achieved in the model by the introduction of a parameter $\mathrm{t_o}$. For collapsed compartments, $\mathrm{t_o}$ is set to τ_c which represents the time it could take for collapsed alveoli to open after a threshold pressure

is reached. Once $p_{trachea} \geq$ TOP$_i$ is satisfied, the counter t_o decrements during every iteration, and triggers the opening of the airway (m_i= 1) as $t_o \leq 0$. Otherwise m_i is set to a high value (10^{10}) to represent a collapsed airway. We based the range of values for TOP used in these simulations on the work done by Crotti and collaborator [19].

N_A (the number of alveolar compartments) is fixed and set by the user (i.e. they do not change during a simulation). Therefore, during a simulation, m_i, chiefly represents the relatively small changes in inlet resistance during tidal ventilation. Furthermore, R_{B0} are also preset and fixed, and do not change during the simulation. The only change in airway resistance which is dynamic is m_i which is dependent on the volume v_i at time(t_k).

Finally, the pulmonary vascular resistance PVR is determined by

$$\frac{1}{PVR} = \frac{1}{R_{V,1}} + \frac{1}{R_{V,2}} + \cdots + \frac{1}{R_{V,N_A}}, \text{for } i = 1, \ldots, N_A \quad (23)$$

where the resistance for each compartment $R_{V,i}$ is defined as

$$R_{V,i} = \delta_{Vi} R_{V0} \quad (24)$$

R_{V0} is the default vascular resistance for the compartment with a value of $160 \cdot N_A$ dynes s cm^{-5} min^{-1}, and δ_{Vi} is the vascular resistance coefficient, used to implement the effect of Hypoxic Pulmonary Vasoconstriction.

The net effect of these components of the simulation is that the defining, clinical features of ARDS may be observed in the model: alveolar gas-trapping (with intrinsic PEEP), collapse-reopening of alveoli (with gradual reabsorption of trapped gas if re-opening does not occur), limitation of expiratory flow etc.

Section 2 - Description of the Cardiac model

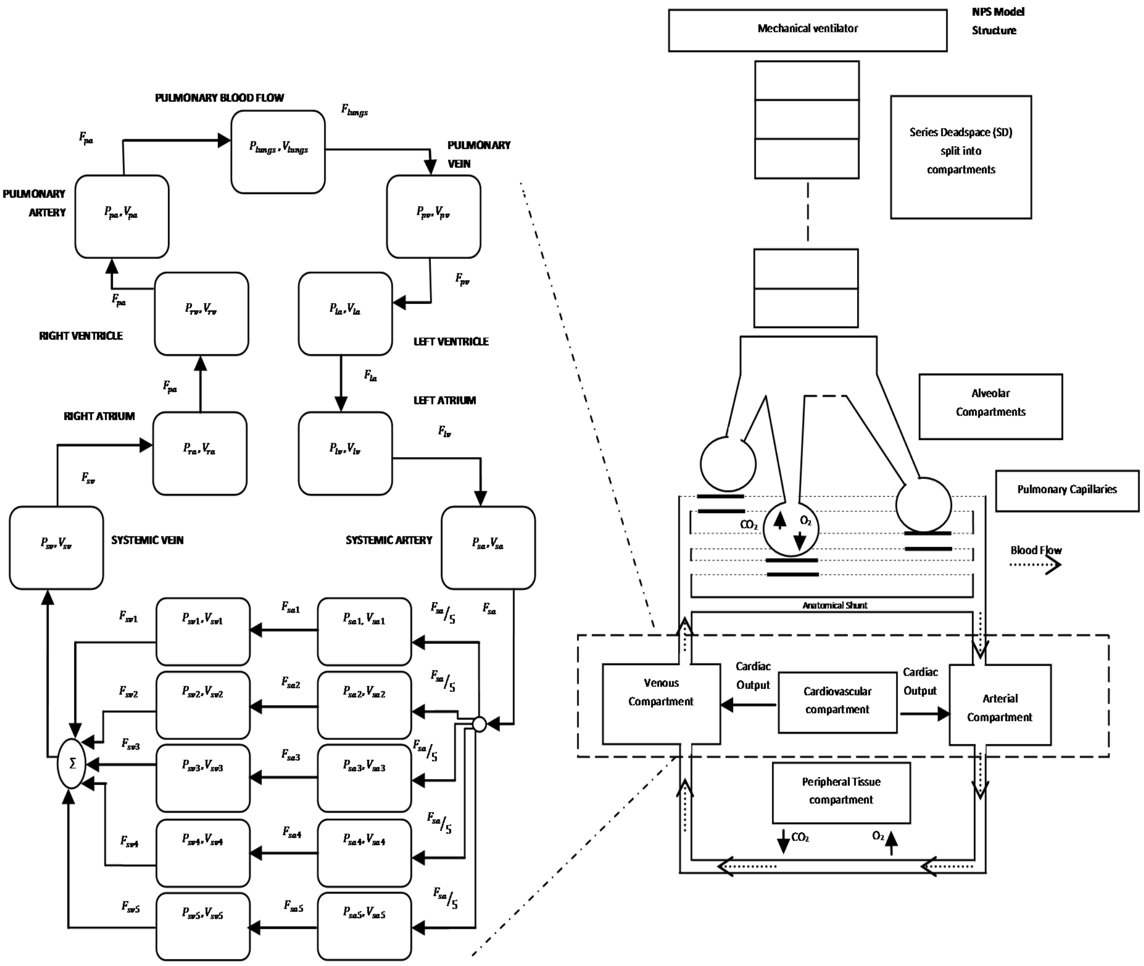

Figure S3: CVS model structure and interaction with pulmonary model

The cardiac model consists of 19 compartments. Each compartment x, is described with a pressure P_x, a volume $\mathrm{V_x}$ and a flow leaving the compartment$\mathrm{F_x}$, which are iteratively updated in a sampling interval. Furthermore, each compartment has the following fixed parameters: a resistance R_x to the flow out of the compartment reflecting the viscosity of the compartment, a coefficient λ_x governing the elastance of each compartment, a coefficient $P_{x,c}$ and $V_{x,u}$, depicting the unstressed volume of the compartment. The ventricles are modeled as having time varying elastances over the duration of a cardiac cycle using different exponential functions to describe the filling and emptying of the ventricles [20]. The shift from the systolic to diastolic relationship is governed by a pulsating activation function with period$T$. For all the compartments, vascular elastance is assumed to be nonlinear and to have an exponential relationship governed by the following equation,

$$P_x = P_{x,c} e^{\frac{\lambda_x (V_x - V_{x,u})}{(V_x + V_{x,u})}} \quad , \tag{25}$$

where the subscript x represents the compartment number and the $\lambda_x, P_{x,c}, V_{x,u}$ are constants that give flexibility in fitting specific shapes and peaks of pressure waveforms that could be observed from clinical data. The model employs separate pressure volume relationships for the systolic and diastolic behavior of ventricles. The left ventricular pressure calculation is given by:

$$P_{lv} = \varphi P_{lv,sys,c} e^{\frac{\lambda_{lv.sys}(V_{lv} - V_{lv,sys,u})}{(V_{lv} + V_{lv,sys,u})}} + (1-\varphi) P_{lv,dys,c} e^{\frac{\lambda_{lv.dys}(V_{lv} - V_{lv,dys,u})}{(V_{lv} + V_{lv,dys,u})}}$$

(26)

The right ventricular pressure calculation is given by:

$$P_{rv} = \varphi P_{rv,sys,c} e^{\frac{\lambda_{rv.sys}(V_{rv} - V_{rv,sys,u})}{(V_{rv} + V_{rv,sys,u})}} + (1-\varphi) P_{lv,dys,c} e^{\frac{\lambda_{rv.dys}(V_{rv} - V_{rv,dys,u})}{(V_{rv} + V_{rv,dys,u})}}$$

(27)

The function φ is a ventricle activation function which is assumed to attain the maximum value of $\varphi = 1$ at the peak of systolic contraction. The function φ attains its minimum value 0 at maximal diastole relaxation. A squared half-sine wave function [20, 21] is adopted for φ given by:

$$\varphi = \begin{cases} \left(\sin\left(\pi T \frac{u}{T_{sys}}\right)\right)^2 & if\ u \geq 0\ and\ u \leq \frac{T_{sys}}{2} \\ 0, & if\ u > \frac{T_{sys}}{2}\ and\ u \leq 1 \end{cases}$$

(28)

$$T = 1/HR, \quad T_{sys} = \frac{(T_{sys,o} - k_{sys})}{T},$$

$$where\ T_{sys,o} = 0.5\ and\ k_{sys} = 0.075$$

(29)

u is a real number ranging between 0 and 1 and it models the fraction of the cardiac cycle. $u = 0$ at the end of systole and $u = 1$ at the end of diastole. T_{sys} indicates the systolic period which is proportional to the heart rate HR (in seconds).

The blood flow between compartments is determined by the pressure gradient between compartments across a linear time invariant resistance R_x.

$$F_x = \frac{\eta_x(P_x - P_y)}{R_x},$$

(30)

$$\eta_x = \begin{cases} 0, & if\ P_x < P_y \\ 1, & if\ P_x \geq P_y \end{cases}.$$

(31)

The parameter η_x allows the blood to flow in one direction but can be altered to investigate flow backwards into a compartment, such as during aortic regurgitation. The volume of the blood in each compartment is computed by applying conservation of mass as follows

$$V_x = (V_{x0} + (F_{\bar{x}} - F_x)\Delta t),$$

(32)

where $F_{\bar{x}}$ is the flow entering the xth compartment (i.e. the flow leaving the upstream compartment) and F_x is the flow leaving the compartment x. V_{x0} is the volume of compartment x before the iteration, and Δt is the size of the time period in this iteration (set to 1 ms). The total amount of blood in the whole body is obtained by $V_T = \sum V_x$.

Section 3 - Cardio pulmonary interactions

The model includes the effect of radial compressive and axial stretching forces exerted onto pulmonary capillaries as a result of increase in lung volume and pressure. The overall effect on resistance to flow through each capillary is difficult to quantify, but we assume the following: (i) at alveolar volumes above the functional residual capacity (FRC), the vessels become compressed and raise the pulmonary vascular resistance (PVR), (ii) at alveolar volumes below FRC, the vessels can collapse and thus result in an increase in PVR, while closer to FRC the PVR remains unaffected. A separate mechanism called hypoxic vasoconstriction, of the vessels contracting in response to hypoxia as a result of alveolar collapse, is already present in the existing pulmonary model. The resultant 'U' shape change in PVR at around the FRC has been suggested previously [22] and has been implemented in this model as follows. The pulmonary vascular resistance PVR is determined as given in equation 23, but the vascular resistance for each alveolar compartment, $R_{V,i}$, is defined has been modified from (24) to

$$R_{V,i} = pvr_{mult,i}\delta_{Vi}R_{V0}. \quad \text{for } i = 1,2,\dots,N_A, \tag{33}$$

N_A is the number of alveolar compartments (set to 100), R_{V0} is the default vascular resistance for the compartment with a value set to $160 \cdot N_A$ dynes s cm^{-5} min^{-1}, and δ_{Vi} is the vascular resistance coefficient, used to implement the effect of Hypoxic Pulmonary Vasoconstriction (and to increase vascular resistance under COPD). $pvr_{mult,i}$ is calculated as follows:

$$pvr_{mult,i} = \left(\left(1 + 0.5\left({(v_i - v_{FRC})}/{v_{FRC}}\right)^2\right)\left(1 + {p_i}/{q_{pvr}}\right)\right)^{n_{pvr}}, \tag{34}$$

where, p_i is the pressure generated within the ith alveolar compartment, v_i is the volume of the ith alveolar compartment, v_{FRC} is a constant representing the volume of the alveolar compartment at rest (fixed to 30 ml). n_{pvr} and q_{pvr} is used to adjust the effect on pulmonary vascular resistance. n_{pvr} is set to 1 and q_{pvr} has been set to 30 but they can be modified to fit patient data.

Increase in lung pressures, such as those observed during the addition of incremental PEEP (Positive End Expiratory Pressure) during ventilation, serves to increase mean intrathoracic pressure to bring about the recruitment and maintenance of collapsed alveolar lung units as mentioned above. In addition to the effect on PVR, the average alveolar compartment pressure within the lung exerts an extrinsic pressure which is applied to the intra-thoracic vascular compartments. This phenomenon is known as splinting. The pressure calculation of the compartments within the thoracic cavity therefore has an additional term, P_{tp}, added to them, representing the intrathoracic pressure

$$P_{tp} = \gamma_{pvr}\left(P_{lungs} - P_{atm}\right). \qquad [35]$$

$\gamma_{pvr} = 0$ for extra-thoracic compartments. Within the thoracic cavity a range of values (0.1-0.8) is used for γ_{pvr} to fit patient data.

Model parameters have been identified from the published literature where available – parameters whose values were not available from the literature were adjusted within their physiological ranges based on the resulting pressure and flow waveforms.

Section 4 - Model calibration to a healthy state

Table S1 lists the data used to get configuration for the CVS model for a Healthy cardio-pulmonary state (from Anesthesia UK: http://www.frca.co.uk/article.aspx?articleid=250). Figure S4 and S5 illustrated the outputs of the cardiopulmonary model in a healthy state. Table S4 lists the values of the parameters used to generate the figures S4 and S5.

Table S1 : Hemodynamic data to prepare the healthy state

Arterial blood pressure (BP) systolic (SBP)	90 - 140 mmHg
Diastolic (DBP)	60 - 90 mmHg
Mean arterial pressure (MAP)	70 - 105 mmHg
Right atrial pressure (RAP)	2 - 6 mmHg
Right ventricular pressure (RVP) systolic (RVSP)	15 - 25 mmHg
Diastolic (RVDP)	0 - 8 mmHg
Pulmonary artery pressure (PAP) systolic (PASP)	15 - 25 mmHg
Diastolic (PADP)	8 - 15 mmHg
Mean pulmonary artery pressure (MPAP)	10 - 20 mmHg
Left atrial pressure (LAP)	6 - 12 mmHg
Cardiac output (CO)	4.0 - 8.0 L/min
Cardiac index (CI)	2.5 - 4.0 L/min/m^2
Stroke volume (SV)	60 - 100 ml/beat
Pulmonary vascular resistance (PVR)	<250 dyne s/cm^5
Right ventricular end-systolic volume (RVESV)	50 - 100 ml
Partial pressure of arterial oxygen (PaO_2)	80 - 100 mmHg
Partial pressure of arterial CO_2 ($PaCO_2$)	35 - 45 mmHg
pH	7.38 - 7.42
Arterial oxygen saturation (SaO_2)	95 - 100%
Mixed venous saturation (SvO_2)	60 - 80%
Oxygen delivery (DO_2)	950 - 1150 ml/min
Oxygen consumption (VO_2)	200 - 250 ml/min

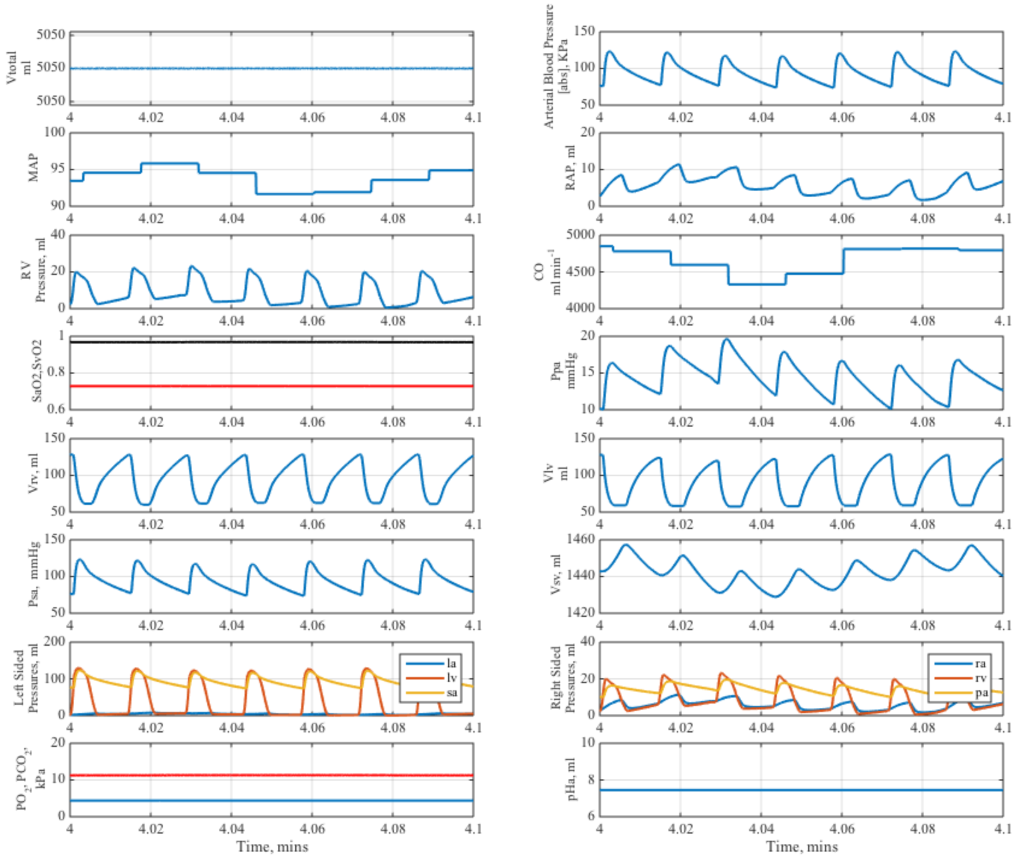

Figure S4: Healthy State model outputs: Vtotal – Total blood volume, MAP – mean arterial blood pressure, RAP, right atrial pressure, RV – righ ventricle, CO – cardiac output, SaO2 – Arterial oxygen saturation, SvO2 – Venous oxygen saturation, Ppa = pulmonary artery pressure, Vrv – volume of right ventricle, Vlv – volume of left ventricle, Psa – systemic artery pressure, Vsv – systemic vein volume, la – left atrium, lv – left ventricle, sa – systemic artery, ra – right atrium, rv – right ventricle, pa – pulmonary artery, PO^2 – arterial partial pressure of oxygen, PCO_2, arterial partial pressure of carbon dioxide, pHa – arterial pH.

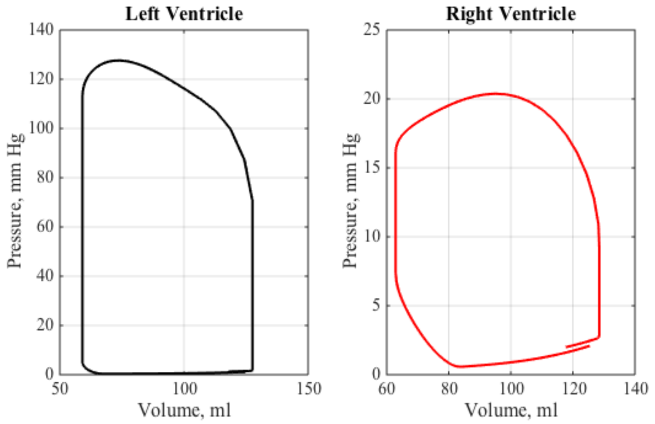

Figure S5: Pressure vs volume of left ventricle (left), and right ventricle (right)

Section 5 - Model calibration to disease state

The complete model calibration to disease configuration algorithm is shown in Table S2.

Table S2: Algorithm for fitting model outputs to patient data in the integrated model

Select model parameters for fitting.<br>Select model parameters for the pulmonary model<br>Select model parameters for the cardiovascular model through sensitivity analysis using Eq. (36).<br>Determine model parameters *x* for pulmonary model<br>Use the pulmonary model, global optimization, and<br>Eq. (37) to determine parameter values (*x*)<br>Determine model parameters for cardiovascular model<br>Use the integrated cardiopulmonary model, global<br>optimization, and Eq. (38), to determine parameter values<br>(*u*) that minimize E_2 at different values of PEEP

Selection of Patient Data

Data regarding three ARDS patients was extracted from three papers from the literature, selected due to their inclusion of data on hemodynamic responses in ARDS patients to changes in mechanical ventilation, specifically changes in cardiac output to variation in positive end expiratory pressure (PEEP). The patients represent a cross section of ARDS patients, with varying severity (using the Berlin definition) and cardiac volemic status. General patient information is listed in Table S2.

The first patient dataset was taken from a paper published by Biondi et al [23]. Based on the data at PEEP = 0 cm H_2O, the patient has a PF ratio of 150 mm Hg and cardiac output (CO) of 8 l min^{-1} (moderate ARDS with high CO). The second patient was taken from data published by Pinsky et al [24] and describes a patient with PF ratio of 167 mm Hg and cardiac output of 4.09 l min^{-1} (moderate ARDS with normal CO). The third patient dataset was taken from data published by Jardin et al [25] and describes a patient with PF ratio of 50 mm Hg and cardiac output of 7.3 l min^{-1} (severe ARDS with high CO).

Assignment of baseline model parameters

To generate a general hemodynamic profile for a healthy subject, most values of resistances, unstressed volumes and pressures have been taken from standard data where available. The remaining model parameters were manually tuned to obtain model outputs within averaged population ranges as given in [26], with the results given in Table S4. The model equations and healthy subject profile are described in detail in [27] and yield a baseline model with which to initiate the model calibration process.

The pulmonary model has already been used to represent data on ARDS patients in [28]. Prior to the calibration of the cardiovascular model, a sensitivity analysis (SA) was performed to identify the model parameters that would dominate the model outputs corresponding to clinical data, which involved calculating *S* below for each model parameter.

$$S = \sum (y_{max} - y_{min}) / y_{baseline} \quad (36)$$

Here, y is model outputs of CO and mean arterial pressure (MAP). y_{max}, y_{min} and $y_{baseline}$ are the maximum, minimum and baseline values of the model outputs calculated during the SA, respectively. A similar sensitivity analysis has earlier shown to be been useful in validating the pulmonary model [6]. The results of the sensitivity analysis indicate key parameters of cardiovascular model (shaded grey in Table S3) in determining CO and MAP. The parameters are consistent with other cardiovascular modelling studies [29].

Model parameter configuration using optimization

The model was configured to fit data from individual ARDS patients in two stages. In the first stage, the model was fitted to static data from separate patients (listed in Table S3). The data consisted of arterial and mixed venous blood gas values and cardiac output estimations for each patient, listing the following measurements at PEEP = 0 cm H_2O: cardiac output in ml min^{-1} (CO), partial pressure of oxygen in arterial blood (PaO_2), partial pressure of carbon dioxide in arterial blood ($PaCO_2$), tidal volume (Vt) and fraction of oxygen inhaled air (F_IO_2). The model parameters (x) to be optimized for each of the 100 alveolar compartments were P_{ext}, k_{stiff} and TOP, representing the extrinsic pressure acting on an alveolar compartment, the stiffness of the compartment and the threshold opening pressure, respectively. The values for respiratory quotient (RQ), rate of breathing (VR), total oxygen consumption (VO_2), hemoglobin levels (Hb), and the inspiratory duty cycle were additional parameters determined by the optimization algorithm. In this case, the model-fitting problem was formulated to search for a configuration of model parameter values (x) that minimizes objective function E_1 in the equation below:

$$\min_x E_1 = \sqrt[2]{\sum_{i=1}^{4} r_j^2} \quad \text{where } r_i = \frac{y_i - y_i'}{y_i'} \tag{37}$$

where y = [PaO_2, $PaCO_2$, TOP_{mean}, P_{peak}] are the model outputs and y' = [PaO_2', $PvCO_2'$, TOP_{mean}', P_{peak}'] are the target values. PaO_2' and $PaCO_2'$ are measurements obtained from the patient data. TOP_{mean} is the average TOP of the alveolar units, which is set to 20 cmH_2O [19]. P_{peak} is the peak airway pressure which is minimized to 30 cm H_2O (a target in the 2000 ARDSnet report [30]).

Stage 2 of the fitting process required a search for the optimal values of the parameters of the cardiovascular models (u) effectively allowing the modification of the cardiovascular function. The optimization process was used to fit the data for changes in CO and mean arterial pressure (MAP) to changes in PEEP. For this stage, the optimization problem was formulated to find a configuration of model parameters (u) that minimizes the objective function E_2:

$$\min_u E_2 = \sqrt[2]{\sum_{j=1}^{k} r_j^2} \quad \text{where } r_j = \frac{y_j - y_j'}{y_j'} \tag{38}$$

where y_j = [CO_i, MAP_j] are the model outputs and y'_j= [CO_i', MAP_i'] are the CO and MAP values reported in the data for the jth PEEP value, with k different settings of PEEP.

The optimization parameters (x) and (u) in stage 1 and stage 2, their sizes, ranges and units are summarized in Table S3. Genetic algorithms (GA's) were employed for the optimization processes of Stage 1 and 2, primarily due to their ease of application in problems with large and small parameter search spaces, and their capability to converge to the global optimum even in highly non-convex parameter spaces. Initial model calibration and analysis were performed on a 64-bit Intel Core i7 3.7

GHz PC, running Matlab (R2014a). Model calibration to data was performed using the ‘Minerva’ high performance computing cluster provided by the University of Warwick (396 nodes, each with 2×hexa-core 2.66 GHz 24 GB RAM) running Matlab (2015a) with global optimization and parallel computing toolboxes.

The parameters shaded grey in Table S3 shows the most sensitive parameters from the SA. Only parameters with S in (Eq.36) of > 5% are listed and selected for Stage 2. Table S3 also shows the results of Stage 1 of the model calibration process. The minimum value calculated for E_1 was 0.3271 for the moderate ARDS high CO patient, 0.3716 for the moderate ARDS normal CO patient and 0.3823 for the severe ARDS high CO patient.

Figure 2 (in the main article) displays the results of stage 2, where the model outputs were matched to increments in PEEP. The minimum value calculated for E_2 was 0.1278 for the moderate ARDS high CO patient, 0.0673 for the moderate ARDS normal CO patient and 0.2172 for the severe ARDS high CO patient.

Finally, Table S4 shows the final parameter configuration of the CVS model for a healthy subject and the three ARDS patients. The parameters in table S4 which are shaded grey are the parameters determined through Stage 2 of model calibration.

Section 6 - List of parameters used for model fitting

Table S3: Parameters and Results of fitting the model to ARDS patient data. N = number of alveolar compartments (100 in this study)

	Parameters	Size	Values set, model outputs or optimization ranges					
			Moderate ARDS, High CO [23]		Moderate ARDS, Normal CO [24]		Severe ARDS, High CO [25]	
Parameters set	CO (l min^{-1})	1	8		4.09		7.3	
	F_iO_2	1	0.5		0.45		1	
	Vt (ml kg^{-1})	1	12		10		10	
	PEEP (cm H_2O)	1	0		0		0	
Ranges of model parameters used for optimization in Stage 1	VR (b min^{-1})	1	10 – 20 *					
	Duty Cycle	1	0.25 - 0.5 *					
	RQ	1	0.7 - 0.9 *					
	VO_2 (ml min^{-1})	1	250 – 350 *					
	TOP (cm H_2O)	N	5 - 70					
	k_{stiff}	N	-1 - 1					
	P_{ext}	N	-30 – 28.8					
	Hb (g dl^{-1})	1	90 – 160 *					
			Data	Model	Data	Model	Data	Model
Results of fitting the data to the model in Stage 1	P_aO_2 (kpa)		10.6	11.2	10	10.8	6.6	7.5
	P_aCO_2 (kpa)		5	4.4	5.3	5.2	3.7	4.3
	PvO2 (kpa)		NA	4.6	NA	4.4	NA	4.1
	Shunt (%)		NA	22	NA	16	NA	44
	TOP_{mean}(cm H_2O)		NA	28	NA	20	NA	29
	P_{peak} (cm H_2O)		NA	32	NA	22	NA	30
Ranges of model parameters used for optimization in Stage 2	$P_{lv,dys,c}$	1	1-5 †					
	$P_{rv,dys,c}$	1	1-5 †					
	$\lambda_{lv,dys,u}$	1	1 - 15 †					
	$\lambda_{lv,sys,u}$	1	1 - 15 †					
	$\lambda_{rv,dys,u}$	1	1 - 15 †					
	$\lambda_{rv,sys,u}$	1	1 - 15 †					
	$\lambda_{sa,u}$	1	1 - 15 †					
	$\lambda_{pa,u}$	1	1 - 15 †					
	$\lambda_{lungs,u}$	1	1 – 15 †					
	$\lambda_{pv,u}$	1	1 - 15 †					
	R_{sa}	1	0.05 - 0.20 †					
	n_{pvr}	1	0.5 - 2 †					
	q_{pvr}	1	40 - 80 †					
	γ_{pvr}	1	0.1 0.8 †					

List of Abbreviations CO - Cardiac Output, F_iO_2 - Fraction of O_2 in inspired gas, Vt - Tidal Volume, VR - Ventilator Rate, PEEP - Positive End Expiratory Pressure, IE - Inspiratory to Expiratory Ratio, RQ - Respiratory Quotient, VO_2 - Oxygen Consumption, TOP - Threshold Opening Pressure, k - alveolar stiffness factor, P_{ext} - Extrinsic pressure, Hb - Hemoglobin in blood, P_aO_2 - Arterial oxygen tension, P_aCO_2 - Arterial carbon dioxide tension, P_vO_2 - Mixed Venous Oxygen tension, Shunt Fraction, $P_{lv,dys,c}$ – Left Ventricle Pressure coefficient, $P_{rv,dys,c}$– Right Ventricle Pressure coefficent, $\lambda_{lv,dys,u}$- left ventricle diastolic elastance, $\lambda_{lv,sys,u}$- left ventricular systolic elastance , $\lambda_{rv,dys,u}$ – right ventricular diastolic elastance, $\lambda_{rv,sys,u}$ – right ventricular systolic elastance, $\lambda_{sa,u}$ – systemic artery elastance, $\lambda_{pa,u}$, pulmonary artery elastance, $\lambda_{lungs,u}$ – elastance of the vascular lung compartment, $\lambda_{pv,u}$ – elastance of pulmonary vein compartment, R_{sa} - systemic arterial resistance, , n_{pvr} – PVR coefficient, q_{pvr}- effect of alveolar compartment coefficient, γ_{pvr} – thoracic pressure coefficient.

* - Optimized results given in main paper Table 1.

† - Optimized results given in Table S4.

Section 7 - Model parameters for simulated patients and healthy state

Table S4: CVS model parameters and their default values for a healthy heart model, the three ARDS patients: Biondi (Moderate ARDS High CO[23]), Pinsky (Moderate ARDS Normal CO [24]), Jardin (Severe ARDS High CO [25]). The shaded parameters were determined in the ARDS patients using optimization (see Table S3 and model fitting section)

Parameter	Symbol	Units	Values			
			Healthy	Biondi	Pinsky	Jardin
Pulmonary Vein Resistance	R_{pv}	mm Hg.s. ml^{-1}	0.0056	0.0056	0.0056	0.0056
Mitral Valve Resistance	R_{la}	mm Hg.s. ml^{-1}	0.008	0.008	0.008	0.008
Aortic Valve Resistance	R_{lv}	mm Hg.s. ml^{-1}	0.01	0.01	0.01	0.01
Systemic Artery Resistance	R_{sa}	mm Hg.s. ml^{-1}	0.14	0.105	0.175	0.10
Systemic Vein Resistance	R_{sv}	mm Hg.s. ml^{-1}	0.0007	0.0007	0.0007	0.0007
Tricuspid Valve Resistance	R_{ra}	mm Hg.s. ml^{-1}	0.001	0.001	0.001	0.001
Pulmonary Valve Resistance	R_{rv}	mm Hg.s. ml^{-1}	0.015	0.015	0.015	0.015
Pulmonary Arterial Resistance	R_{pa}	mm Hg.s. ml^{-1}	0.005	0.005	0.005	0.005
Total blood Volume	V_T	ml	5050	5050	5050	5050
Volume in Cardiac Chambers	V_H	ml	0.066 V_T	0.066 V_T	0.066 V_T	0.066 V_T
Left ventricle systolic volume constant	$V_{lv,sys,u}$	ml	0.32 V_H	0.32 V_H	0.32 V_H	0.32 V_H
Left ventricle diastolic volume constant	$V_{lv,dys,u}$	ml	$V_{lv,sys,u} - 40$	$V_{lv,sys,u} - 40$	$V_{lv,sys,u} - 40$	$V_{lv,sys,u} - 40$
Right ventricle systolic volume constant	$V_{rv,dys,u}$	ml	0.38 V_H	0.38 V_H	0.38 V_H	0.38 V_H
Right ventricle diastolic volume constant	$V_{rv,sys,u}$	ml	$V_{rv,sys,u} - 40$	$V_{rv,sys,u} - 40$	$V_{rv,sys,u} - 40$	$V_{rv,sys,u} - 40$
Right Atrium, unstressed volume	$V_{ra,u}$	ml	0.15 V_H	0.15 V_H	0.15 V_H	0.15 V_H
Left Atrium, unstressed volume	$V_{la,u}$	ml	0.15 V_H	0.15 V_H	0.15 V_H	0.15 V_H
Total Systemic Arterial volume	VSA	ml	0.24 V_T	0.24 V_T	0.24 V_T	0.24 V_T
Systemic Artery unstressed volume	$V_{sa,u}$	ml	0.5 VSA	0.5 VSA	0.5 VSA	0.5 VSA
Systemic Arterioles unstressed volume	$V_{sai,u}$	ml	0.1 VSA	0.1 VSA	0.1 VSA	0.1 VSA
Total Systemic Venous volume	VSV	ml	0. 60 V_T	0. 60 V_T	0. 60 V_T	0. 60 V_T
Systemic Vein unstressed volume	$V_{svi.u}$	ml	0.65 VSV	0.65 VSV	0.65 VSV	0.65 VSV
Systemic Venules unstressed volume	$V_{sv,u}$	ml	0.07 VSV	0.07 VSV	0.07 VSV	0.07 VSV
Pulmonary Artery unstressed volume	$V_{pa,u}$	ml	0.023 V_T	0.023 V_T	0.023 V_T	0.023 V_T
Pulmonary compartment unstressed volume	$V_{lungs,u}$	ml	0.013 V_T	0.013 V_T	0.013 V_T	0.013 V_T
Pulmonary Vein unstressed volume	$V_{pv,u}$	ml	0.054 V_T	0.054 V_T	0.054 V_T	0.054 V_T
Coefficient for end diastolic pressure in left ventricle	$P_{lv,dys,c}$	mm Hg	2	2	3	1
Coefficient for end systolic pressure in left ventricle	$P_{lv,sys,c}$	mm Hg	110	110	110	110
Coefficient for end diastolic pressure in right ventricle	$P_{rv,dys,c}$	mm Hg	2	1	3	3
Coefficient for end systolic pressure in right ventricle	$P_{rv,sys,c}$	mm Hg	12	12	12	12
Coefficient for relaxed left atrium	$P_{la,c}$	mm Hg	5	5	5	5
Coefficient for relaxed right atrium	$P_{ra,c}$	mm Hg	5	5	5	5
Coefficient for relaxed systemic artery	$P_{sa,c}$	mm Hg	110	110	110	110
Coefficient for relaxed systemic vein	$P_{sv,c}$	mm Hg	8	8	8	8
Coefficient for relaxed systemic arterioles	$P_{sai,c}$	mm Hg	20	20	20	20
Coefficient for relaxed systemic venules	$P_{svi.c}$	mm Hg	1.6	1.6	1.6	1.6
Coefficient for relaxed pulmonary artery	$P_{pa,c}$	mm Hg	15	15	15	15
Coefficient for relaxed lung compartment	$P_{lungs,c}$	mm Hg	13	13	13	13
Coefficient for relaxed pulmonary vein	$P_{pv,c}$	mm Hg	12	12	12	12
Coefficient for elastance of left ventricle diastole	$\lambda_{lv,dys,u}$	-	10	15	9	10
Coefficient for elastance of left ventricle systole	$\lambda_{lv,sys,u}$	-	5	9	11	1
Coefficient for elastance of right ventricle diastole	$\lambda_{rv,dys,u}$	-	10	15	9	10
Coefficient for elastance of right ventricle systole	$\lambda_{rv,sys,u}$	-	5	9	11	1
Coefficient for elastance of left atrium	$\lambda_{la,u}$	-	9	9	9	9
Coefficient for elastance of right atrium	$\lambda_{ra,u}$	-	9	9	9	9
Coefficient for elastance of systemic artery	$\lambda_{sa,u}$	-	10	12	11	10
Coefficient for elastance of systemic arterioles	$\lambda_{sai,u}$	-	10	10	10	10
Coefficient for elastance of systemic venules	$\lambda_{svi.u}$	-	10	10	10	10
Coefficient for elastance of systemic vein	$\lambda_{sv,u}$	-	10	10	10	10
Coefficient for elastance of pulmonary artery	$\lambda_{pa,u}$	-	5	9	9	10
Coefficient for elastance of vascular lung compartment	$\lambda_{lungs,u}$	-	5	5	9	10
Coefficient for elastance of pulmonary vein	$\lambda_{pv,u}$	-	11	5	9	10
Splinting coefficient	γ_{pvr}	mm Hg	0.5	0.4	0.5	0.5
PVR exponent	n_{pvr}	-	1	1	1	0.5
PVR coefficent	q_{pvr}	-	60	80	80	60

Section 8 – Other key hemodynamic and pulmonary parameters

MRS

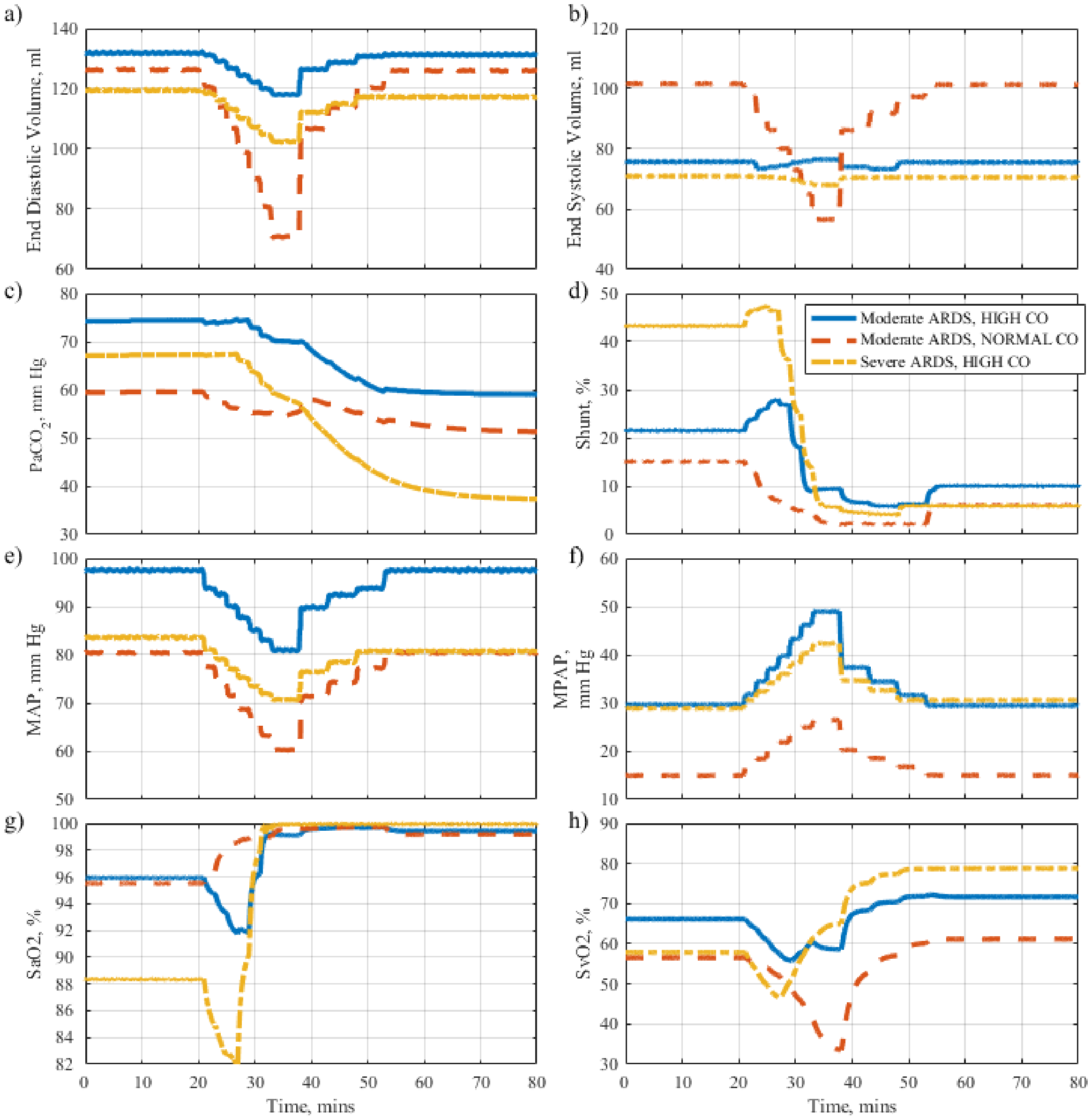

Figure S6: Results of applying the maximum recruitment strategy (MRS) to three ARDS patients. Plots of: a) and b) right ventricle volume end diastolic and end systolic volume, c) arterial carbon dioxide tension ($PaCO_2$), d) physiological shunt (Shunt), e) mean arterial pressure (MAP), f) mean pulmonary arterial pressure (MPAP), g) arterial oxygen saturation (SaO_2) and h) venous oxygen saturation (SvO_2).

SI

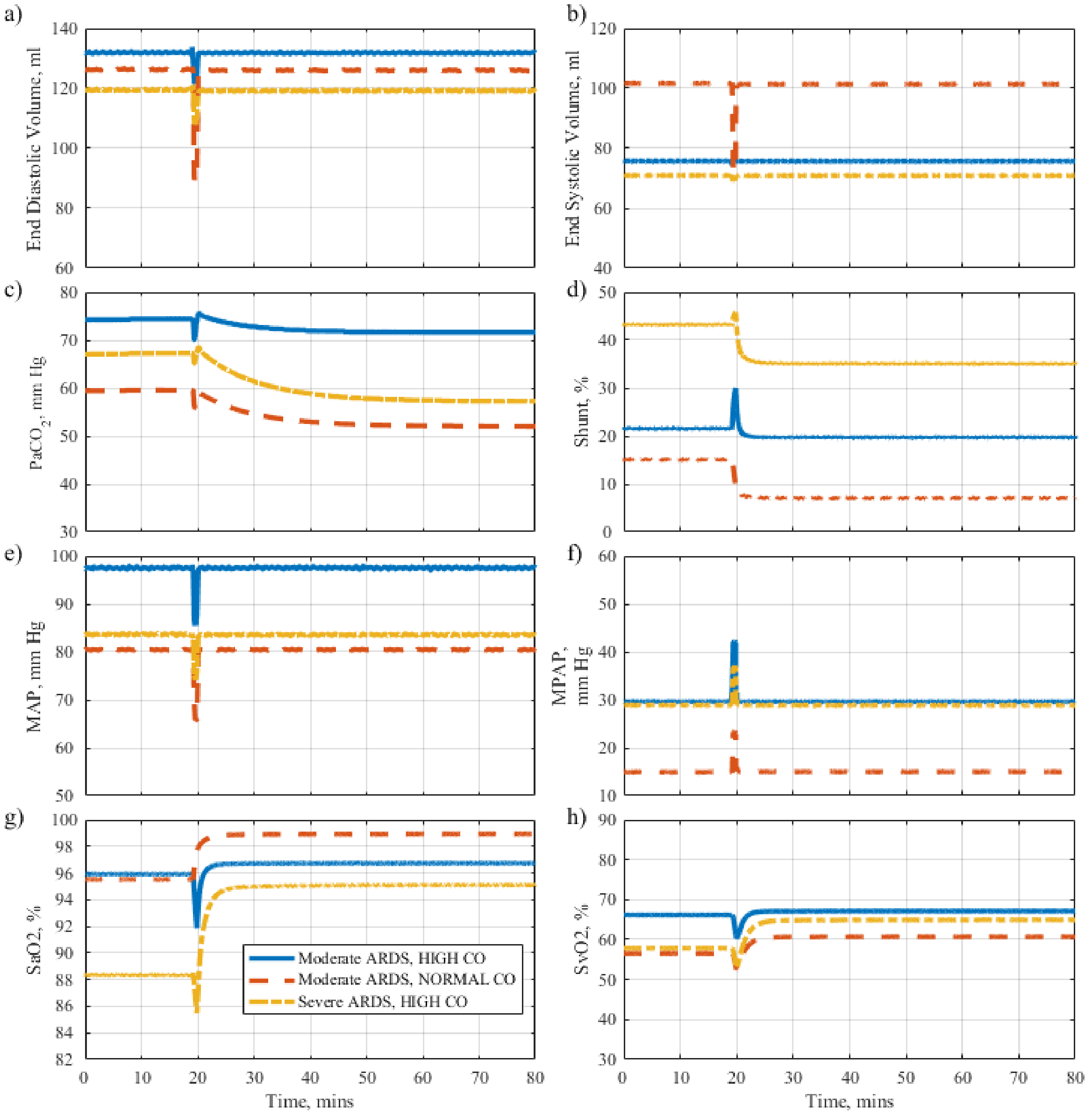

Figure S7: Results of applying the Sustained Inflation (SI) to three ARDS patients. Plots of: a) and b) right ventricle volume end diastolic and end systolic volume, c) arterial carbon dioxide tension ($PaCO_2$), d) physiological shunt (Shunt), e) mean arterial pressure (MAP), f) mean pulmonary arterial pressure (MPAP), g) arterial oxygen saturation (SaO_2) and h) venous oxygen saturation (SvO_2).

References

1. Hardman J, Aitkenhead A: **Estimation of alveolar deadspace fraction using arterial and end-tidal CO2: a factor analysis using a physiological simulation**. *Anaesthesia and intensive care* 1999, **27**(5):452.
2. Hardman J, Bedforth N: **Estimating venous admixture using a physiological simulator**. *British journal of anaesthesia* 1999, **82**(3):346-349.
3. Hardman J, Bedforth N, Ahmed A, Mahajan R, Aitkenhead A: **A physiology simulator: validation of its respiratory components and its ability to predict the patient's response to changes in mechanical ventilation**. *British journal of anaesthesia* 1998, **81**(3):327-332.
4. Hardman JG, Aitkenhead AR: **Validation of an original mathematical model of CO2 elimination and dead space ventilation**. *Anesthesia & Analgesia* 2003, **97**(6):1840-1845.
5. Hardman J, Wills J: **The development of hypoxaemia during apnoea in children: a computational modelling investigation**. *British journal of anaesthesia* 2006, **97**(4):564-570.
6. Das A, Gao Z, Menon P, Hardman J, Bates D: **A systems engineering approach to validation of a pulmonary physiology simulator for clinical applications**. *Journal of The Royal Society Interface* 2011, **8**(54):44-55.
7. McCahon R, Columb M, Mahajan R, Hardman J: **Validation and application of a high-fidelity, computational model of acute respiratory distress syndrome to the examination of the indices of oxygenation at constant lung-state**. *British journal of anaesthesia* 2008, **101**(3):358-365.
8. Hardman JG, Al-Otaibi HM: **Prediction of arterial oxygen tension: validation of a novel formula**. *American journal of respiratory and critical care medicine* 2010, **182**(3):435-436.
9. Marshall BE, Clarke WR, Costarino AT, Chen L, Miller F, Marshall C: **The dose-response relationship for hypoxic pulmonary vasoconstriction**. *Respir Physiol* 1994, **96**(2-3):231-247.
10. Severinghaus JW: **Simple, accurate equations for human blood O2 dissociation computations**. *J Appl Physiol Respir Environ Exerc Physiol* 1979, **46**(3):599-602.
11. Severinghaus JW: **Blood gas calculator**. *J Appl Physiol* 1966, **21**(3):1108-1116.
12. Douglas AR, Jones NL, Reed JW: **Calculation of whole blood CO2 content**. *J Appl Physiol (1985)* 1988, **65**(1):473-477.
13. McHardy GJ: **The relationship between the differences in pressure and content of carbon dioxide in arterial and venous blood**. *Clin Sci* 1967, **32**(2):299-309.
14. Kelman GR, Nunn JF: **Nomograms for correction of blood Po2, Pco2, pH, and base excess for time and temperature**. *J Appl Physiol* 1966, **21**(5):1484-1490.
15. Siggaard-Andersen O: **The van Slyke equation**. *Scand J Clin Lab Invest Suppl* 1977, **146**:15-20.
16. Lachmann B: **Open up the lung and keep the lung open**. *Intensive Care Med* 1992, **18**(6):319-321.
17. Hickling KG: **The pressure-volume curve is greatly modified by recruitment. A mathematical model of ARDS lungs**. *Am J Respir Crit Care Med* 1998, **158**(1):194-202.
18. Bates JH, Irvin CG: **Time dependence of recruitment and derecruitment in the lung: a theoretical model**. *J Appl Physiol (1985)* 2002, **93**(2):705-713.
19. Crotti S, Mascheroni D, Caironi P, Pelosi P, Ronzoni G, Mondino M, Marini JJ, Gattinoni L: **Recruitment and derecruitment during acute respiratory failure: a clinical study**. *Am J Respir Crit Care Med* 2001, **164**(1):131-140.
20. Ursino M: **Interaction between carotid baroregulation and the pulsating heart: a mathematical model**. *American Journal of Physiology-Heart and Circulatory Physiology* 1998, **275**(5):H1733-H1747.
21. Piene H: **Impedance matching between ventricle and load**. *Annals of biomedical engineering* 1984, **12**(2):191-207.
22. Shekerdemian L, Bohn D: **Cardiovascular effects of mechanical ventilation**. *Archives of disease in childhood* 1999, **80**(5):475-480.
23. Biondi JW, Schulman DS, Soufer R, Matthay RA, Hines RL, Kay HR, Barash PG: **The effect of incremental positive end-expiratory pressure on right ventricular hemodynamics and ejection fraction**. *Anesth Analg* 1988, **67**(2):144-151.
24. Pinsky MR, Desmet JM, Vincent JL: **Effect of positive end-expiratory pressure on right ventricular function in humans**. *Am Rev Respir Dis* 1992, **146**(3):681-687.
25. Jardin F, Farcot JC, Boisante L, Curien N, Margairaz A, Bourdarias JP: **Influence of positive end-expiratory pressure on left ventricular performance**. *N Engl J Med* 1981, **304**(7):387-392.
26. Tables GS: **Volume 5, Heart and Circulation**. *Basel Switze rlan d elBA-GEIGY Limited* 1990.
27. Das A, Haque M, Cole O, Chikhani M, Wang W, Ali T, Bates DG, Hardman JG: **Development of an Integrated Model of Cardiovascular and Pulmonary Physiology for the Evaluation of Mechanical Ventilation Strategy**. In: *37th IEEE Engineering in Medicine and Biology Conference , Milano, Italy: 2015; Milano, Italy*; 2015.
28. Das A, Cole O, Chikhani M, Wang W, Ali T, Haque M, Bates DG, Hardman JG: **Evaluation of lung recruitment maneuvers in acute respiratory distress syndrome using computer simulation**. *Critical Care* 2015, **19**(1):8.
29. Liang F, Sughimoto K, Matsuo K, Liu H, Takagi S: **Patient-specific assessment of cardiovascular function by combination of clinical data and computational model with applications to patients undergoing Fontan operation**. *Int J Numer Method Biomed Eng* 2014, **30**(10):1000-1018.
30. De Campos T: **Ventilation with lower tidal volumes as compared with traditional tidal volumes for acute lung injury and the acute respiratory distress syndrome. The Acute Respiratory Distress Syndrome Network**. *N Engl J Med* 2000, **342**(18):1302-1308.
